# A pleiotropic QTL increased economic water use efficiency in bread wheat (*Triticum aestivum* L.)

**DOI:** 10.3389/fpls.2022.1067590

**Published:** 2023-01-04

**Authors:** Jian Hui, Haibo Bai, Xuelian Lyu, Sishuang Ma, Xiaojun Chen, Shuhua Li

**Affiliations:** Ningxia Key Laboratory of Agricultural Biotechnology, Agricultural Biotechnology Research Center, Ningxia Academy of Agriculture and Forestry Science, Yinchuan, Ningxia, China

**Keywords:** water use efficiency of economic yield, carbon isotope discrimination, QTL, recombinant inbred lines, bread wheat (*Triticum aestivum* L.)

## Abstract

Wheat is one of the most important food crops in the world and drought can severely impact on wheat productivity. The identification and deployment of genes for improved water use efficiency (WUE) can help alleviate yield loss under water limitation. In this study, a high-density genetic linkage map of wheat recombinant inbred lines (Ningchun 4 x Ningchun 27) containing 8751 specific locus amplified fragment (SLAF) tags (including 14757 SNPs), with a total map distance of 1685 cM and an average inter-marker map distance of 0.19 cM was constructed by SLAF-seq technology. The economic yield WUE and nine related traits under three water treatments was monitored over four years. The results showed that loci conditioning WUE were also associated with grain carbon isotope discrimination (CID), flag leaf chlorophyll content, plant height, 1000-grain weight, grain weight per spike and grain number per spike. One locus on chromosome 2B explained 26.3% WUE variation in multiple environments. Under good soil moisture conditions before flowering, the high CID genotype *QWue.acn-2B* ^Ningchun 27^, was associated with WUE, high grain weight per spike, and kilo-grain weight. Under rain-fed conditions, the low CID genotype *QWue.acn-2B* ^Ningchun 4^ tended to maintain more spike number and was associated with improved WUE and yield. The introduction of good chromosome fragments of *QWue.acn-2B* into elite lines by molecular marker assisted selection will boost up the cultivation of high-yield and water-saving wheat varieties.

## Introduction

Wheat, as a staple food for about 40% of the world’s population, is one of the most important food crops, with the largest sown area and the widest distribution in the world (Acevedo et al., 2018). Drought is an increasingly important environmental factor restricting the productivity of wheat. With diminishing water resources for irrigation, improving yield under drought has become the main breeding goal for many crops (Sivamani et al., 2000). Breeding wheat varieties with high water use efficiency of economic yield is a major measure to increase productivity, especially under water-limited conditions (Ehdaie and Waines, 1993). At present, the cultivation of water-efficient varieties is generally carried out from two aspects: Leaf WUE or Population WUE and related agronomic traits (such as plant height, yield, and yield related traits) (Hsiao, 2000; Narasimhamoorthy et al., 2006; Kuneg et al., 2007). For leaf WUE, the two main parameters are considered, including intrinsic water use efficiency (WUE_i_) and instantaneous water use efficiency (WUE_inst_). The former is the ratio of net photosynthetic rate (*P_n_*) to stomatal conductance (*g_s_*), which is used to evaluate the effect of genotype on plants, while the latter is the ratio of leaf *P_n_* to transpiration rate (*Tr*), which is mainly used to estimate the impact of environmental factors on plants (Fischer and Turner, 1978; Gago et al., 2014; Franks et al., 2015; Khler et al., 2015). Population WUE was divided into biological yield WUE (Biomass/Total water consumption during growth period) and economic yield WUE (Economic yield/Total water consumption during growth period). From the perspective of breeding and agricultural production, screening and utilization of WUE genotypes with higher economic yield is a powerful means to address the contradiction between drought and high yield.

Drought induces a series of adaptive changes in the structure, physiology and biochemistry of cells in crops, resulting in differences in growth and development, whereas some morphological changes can affect economic WUE and yield. It has been reported that grain filling rate, grain yield, number of grains per ear, and 1000-grain weight under drought stress are effective indicators for the identification of crop drought resistance and water use efficiency (Li and Pan, 2005). There was a significant positive correlation between wheat canopy temperature depression below air temperature and stomatal conductance and yield (Amani et al., 2010), and under variable field conditions, the SPAD (Soil and plant analysis development) value of flag leaves was significantly positively correlated with yield (Islam et al., 2014). SPAD value and canopy temperature can be useful indicators for drought stress tolerance (Srivastava et al., 2017) of different wheat genotypes (Balota et al., 1999), and can also be used to predict wheat WUE and yield. For wheat, dwarf genes can enhance wheat lodging resistance, improve harvest index (Richards, 1992), and indirectly affect WUE. Previous studies have found that *Rht1*, *Rht2* and *Rht3* genes have negative effects on WUE (Ehdaie and Waines, 1994), while *Rht13* and *Rht8* genes have positive effects on WUE (Yan and Zhang, 2017). Currently, carbon isotope discrimination (Δ or CID) is widely used as an indirect assessment of the water status of C3 crops under water-limited conditions. A negative correlation between Δ and WUE was confirmed in multiple C3 species (Farquhar and Richards, 1984; Hubick et al., 1986; Hubick and Farquhar, 1989; Condon et al., 1990; Hall et al., 1990; Hall et al., 1992; Ismail and Hall, 1992; Rebetzke et al., 2002; Lambrides et al., 2004; Rytter, 2005). However, this conclusion is usually obtained in pot conditions or at the level of single plant and single leaf, and is less relevant to the relationship between Δ, WUE and yield at the population level. In fact, WUE of leaf does not always translate into higher crop WUE or yield (Condon et al., 2004). CID and grain yield differ depending on material source, tissue site, sampling time and water status can be positive, neutral or negative (Condon et al., 2002; Monneveux et al., 2005; Xing et al., 2007; Khazaei and Mohammady, 2010). It has been reported that Δ usually decreases from the oldest part of the plant to the youngest part under different water conditions (Hubick and Farquhar, 1989; Acevedo, 1993), and the mature grain is the most suitable part for measuring Δ. Compared with the leaf of Δ, the grain of Δ has higher generalized heritability, better correlation with harvest index and grain yield, can reflect the efficiency of carbon allocation to grain, and well predict yield (Merah et al., 2001).

Molecular biology and genomics have become major tools for dissecting trait potential and unearthing genes for trait improvement (Kole et al., 2015; Henry et al., 2018). Analysis of shoot δ13C (a natural isotope of carbon) of the Chinese Spring wheat-Betzes barley disomic addition line indicated that a gene controlling WUE was carried on chromosome 4H (Handley et al., 1994). A study of “chinese spring” group D chromosome substitution lines showed that the wheat chromosome 7D increased WUE (Gorny, 1999). Using Δ as as an indicator of WUE, the QTL controlling transpiration efficiency was mapped on the *ERECTA* marker on chromosome 2, and the *ERECTA* gene was cloned from *Arabidopsis thaliana*, it regulates transpiration efficiency by changing leaf stomatal number and leaf structure (Masle et al., 2005). It has been reported that genes associated with high photosynthetic rate and high WUE were detected on chromosome arms 1AL, 2AL, 2AS and 7AS, In addition, 2AL had a gene that controlled a low transpiration rate (Zhang and Shan, 2000). Three wheat mapping populations grown under well-watered conditions were used to identify identical or overlapping loci for numerous quantitative trait loci (QTL) for canopy temperature and leaf porosity and plant height (Rebetzke et al., 2013). These research studies provide a reference for utilization of high WUE genes for drought resistance in wheat, but they do not specify the impact of genes/QTL on economic yield.

QTL mapping studies can be used to identify molecular markers closely linked with functional genes and even the functional genes themselves, which provides a shortcut for molecular marker-assisted (MAS) breeding of high WUE materials (varieties) (Pinto et al., 2010; Sun et al., 2017; Yadav et al., 2022). Amongst molecular markers, SNPs markers are the most numerous, widely distributed and highly polymorphic, allowing the construction of high-density genetic maps that are conducive to the accurate positioning of QTLs. SLAF-seq is a high-throughput sequencing-based method for large-scale genotyping (Sun et al., 2013), which can obtain DNA fragments of specific length (SLAF tags) by restriction enzyme digestion. The advantages of this method include high throughput, high accuracy, low cost, and short cycle and has been successfully applied to many species such as crops, vegetables, forest trees and aquatic products (Zhang et al., 2013; Wei et al., 2014; Zhu et al., 2016; Hu et al., 2016; Yin et al., 2018; Zhang et al., 2020; Wei et al., 2020). At present, there are few reports on population-level mapping of wheat economic water use efficiency traits. In this study, SLAF-seq technology was used to construct a high-density genetic map of wheat recombinant inbred lines (RIL) that were evaluated for agronomic WUE-related phenotypes over multiple years.

## Materials and methods

### Plant materials

The F_10:11_ recombinant inbred lines (n=128) were derived from a cross between bread wheat Ningchun 4 and Ningchun 27. Ningchun 4 is the main variety in Ningxia Yellow River irrigation area, with high and stable yield and Ningchun 27 is the main variety planted in the dry land in the mountainous area of Southern Ningxia, with strong drought tolerance. They are the backbone materials of wheat breeding in Ningxia province, China. The former has higher WUE under irrigation conditions and lower WUE under dry farming conditions, and the latter is just the opposite. Genomic DNA for the SLAF-seq was extracted from young leaves at seedling stage of parents and RILs using the cetyltrimethylammonium bromide (CTAB) method.

### Test design

The experiments were conducted in the Ningxia irrigation area (Yongning County: 1144 m above sea level, 150-200 mm annual rainfall, 1700-1900 mm evaporation, and less than 100 mm rainfall during the growth period of spring wheat), and the Ningxia rain fed area (Yuanzhou District: altitude 1567 m, annual rainfall 300-400 mm, evaporation 1700-1900 mm, rainfall 180-300 mm during the growth period of spring wheat). The climatic characteristics of the test site in the wheat growing season are shown in **Supplementary Table S1**. RILs were planted in plots, with 6 rows in each plot, a length of 3 m, a row spacing of 0.15 m, and a plot area of 2.7 m^2^. The field trial design used randomized complete block design. RILs were randomly arranged in each trial treatment and repeated twice.

Three treatments were designed among which two (T1 & T2) were irrigated and one (T3) was rainfed.Two water treatments were set in the irrigated area: normal irrigation (T1): irrigated a total of four times during the growth period at the tillering, jointing, heading and filling stages with 1200 m^3^/ha, 900 m^3^/ha, 900 m^3^/ha and 600 m^3^/ha, respectively. The irrigation quota was 3600 m^3/^ha. Water stress treatment after heading (T2): the plots were irrigated as described above the tillering and jointing stages (total irrigation of 2100 m^3^/ha). A water measuring weir was used to measure and control the amount of irrigation water. There was a protection area more than 5 m around the test area, and the agricultural film was pressed into a depth of 2 m to prevent lateral water leakage. In rain fed areas, no irrigation was used during the whole wheat growth period: rain-fed only (T3). Field irrigation amount of wheat under different treatments were listed in **Supplementary Table S2**.

### Measurements of agronomic and physiological traits

Traits and measurement time are presented in **Supplementary Table S3**. The specific measurement methods of traits are as follows.

CT measurements: 15 days after flowering (milky stage), Model 2958 infrared thermometer (Spectrum Technologies Inc.) was used to measure the temperature of the wheat canopy in each plot three times from 13:00 to 15:00 in the afternoon of sunny days, and the mean value was recorded.

LCC measurements: 15 days after flowering (milky stage), the flag leaves of 10 wheat plants randomly selected from each plot were measured with a SPAD-502 chlorophyll meter (Spectrum Technologies Inc.), and the mean value was recorded.

SNUA and GYUA measurements: the number of spikes in the middle two rows of each plot at maturity was determined, and this was converted to the number of spikes per hectare. After harvest, plot yields were determined and converted into yield per hectare.

PH, FSN, KGW, GWS and GNS measurements: 10 plants were sampled from each plot at the maturity stage for testing, and the average value of each agronomic trait was taken. WUE calculation: grain yield/water consumption (ET) was used to calculate the economic yield and WUE of each variety for each treatment, and the water balance method was used to estimate ET, ET= (WH + R + E) - (WK+ F + N), where ET is crop water consumption; WH is the water storage in a two meters soil layer at the time of sowing; R is the sum of rainfall and irrigation during the growth period; E is the amount of water entering a two meters soil layer from the lower soil layer; WK is the water storage in two meters soil layer at harvest; F is the amount of water penetrating below two meters; N is runoff.

CID measurements: at maturity, 20 plants were randomly calibrated for each line, and the mixed grains were used as the samples to be tested. After the samples were washed with distilled water, they were dried at 70 °C for 48 hours to a constant weight, ground into a 100 mesh sieve, and put into a 1.5ml centrifuge tube. Bast (Beijing) Institute of desertification control technology was entrusted to determine the carbon isotopic composition of wheat samples with mat XP isotope mass spectrometer (δ^13^C), with PDB (PEE Dee belemnite) as the standard. δ^13^C formula is calculated as follows: δ^13^C (‰) = [(R_sample/_R_PDB_ - 1) × 1000] (R is ^13^C/^12^C ratio), and the calculation formula of carbon parity discrimination (△ or CID) is: △ (‰) = [(δ^13^C_air_ - δ^13^C_sample_) ×1000]/(1 + δ^13^C_sample_), where, δ^13^C_air_ = - 8‰ (Farquhar et al., 1989).

### QTL analysis

SLAF-seq analysis was conducted by Biomarker Technologies Corporation (Address:12 Fuqian Street, Shunyi District, Beijing, China). The library was sequenced using the Illumina HiSeq platform. A high-density genetic map was drawn by HighMap software (Li et al., 2008). Multi-environmental traits were mapped using the additive effect model (ICIM-ADD) of IciMapping 4.2 software for complete interval mapping, and the QTL mapping function was used to convert the recombination rate to genetic distance (cM) using the Kosambi mapping function with a scan step size of 1 cM, a LOD critical value of 2.5, and using statistical detection threshold of P=0.001. Standard naming of QTL in wheat according to the standard nomenclature described in the Cataloque of Gene Symbols for wheat (Mcintosh et al., 2013), for example, *QPh.acn-4D.1* was the first QTL associated with plant height on chromosome 4D, which was detected by Agricultural Biotechnology Research Center of Ningxia (ACN). Major QTLs were defined as those having a phenotypic variation explanation rate (PVE) of greater than 10%. The relative position and genetic positions of QTLs on chromosomes were drawn by R 4.1.0 and MG2C_v2.1 (Chao et al., 2021), respectively.

### Data processing

A mixed linear model was used, with year as a random factor, and strain and treatment as fixed factors, to obtain the best linear estimator (BLUE value) of WUE of each variety and its main related traits. R studio version 4.1 was used to plot WUE and to generate the correlation heatmap of the main agronomic and physiological traits.

## Results

### Genetic map construction

**Figure 1:**
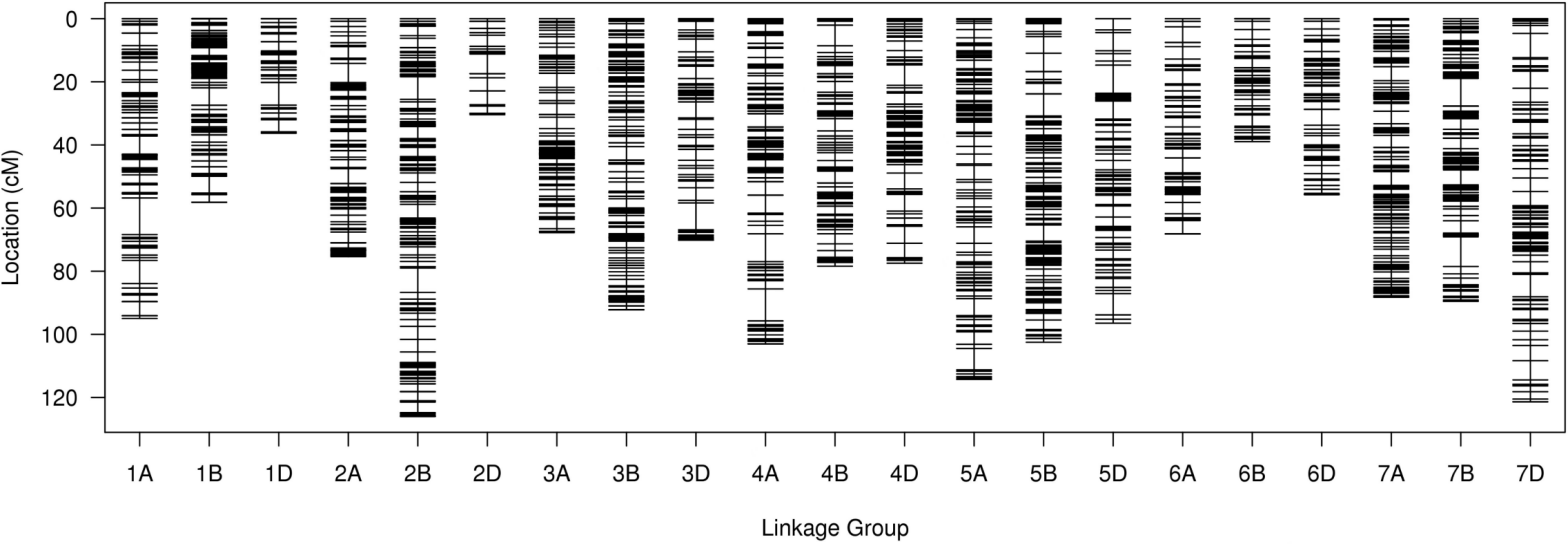
Genetic linkage map of wheat.

For the SLAF-seq analysis, the average sequencing depth of parents was 18.8X, including 20.0X for Ningchun 4, 17.6X for Ningchun 27 and 6.3X for the offspring. A total of 1,270,454 SLAF tags were developed, including 186,087 polymorphic SLAF tags, with a polymorphism ratio of 14.65%. A total of 89968 (aa×bb type) SLAF tags suitable for wheat RILs were developed. After removing markers with low quality, low depth, low coverage and very significant partial separation, 8751 SLAF tags (including 14,757 SNPs) were finally obtained for genetic map construction. By comparing with the reference genome (IWGSC RefSeq v1.0: https://urgi.versailles.inra.fr/download/iwgsc/IWGSC_RefSeq_Annotations/v1.0/), the SLAF tags were divided into 21 linkage groups representing the 21 wheat chromosomes. The linear arrangement of markers in the linkage group was analyzed by HighMap software, and the genetic distance between adjacent markers was estimated. Finally, a genetic map with a total map distance of 1685.32 cM and an average map distance of 0.19 cM was obtained (**Figure 1**). The number of linkage group markers ranged from 53 (2D) to 1020 (2A), and the average genetic distance ranged from 0.06 cM (1B) to 0.69 cM (7D). The lowest proportion of gap length less than 5 cM in the linkage group was 97.73% (7D), the highest was 100% (3A, 3B, 6A, 6B and 7A), and the maximum gap was between 11.62 cM (1A) and 3.58 cM (6B). A total of 194 markers (2.2% of the total) with partial segregation were included in the construction of the map. The proportion of double exchange of markers in each linkage group was very low, ranging from 0.02% (1B) to 0.39% (5B), and the proportion of deletions ranged from 1.19% (1B) to 18.19% (6B). Spearman rank correlation coefficient revealed that the correlation coefficient between the sequence of markers of each linkage group of the determined map and their position in the reference genome is high, indicating that the map is of high quality. The marker number, total map distance, average map distance, gap < 5 cM ratio, maximum gap, the number of partial segregation markers, double exchange ratio, deletion rate, and collinearity correlation coefficient of each linkage group are shown in **Table 1**.

**Table 1:** Basic statistics of the genetic map.

Chromosome ID	SLAF Number	Total Distance (cM)	Average Distance (cM)	Gaps <=5(%)	Max Gap	BS	SP (%)	Miss (%)	Spearman
1A	348	94.95	0.27	99.14	11.62	8	0.21	11.62	1.00
1B	999	58.15	0.06	99.80	5.52	0	0.02	1.19	0.90
1D	110	36.26	0.33	99.08	7.19	0	0.43	9.19	0.99
2A	1020	75.39	0.07	99.90	6.05	4	0.21	8.83	0.97
2B	873	125.99	0.14	99.77	7.71	0	0.18	9.84	0.89
2D	53	30.40	0.58	98.08	6.22	17	0.31	14.24	0.91
3A	251	67.79	0.27	100.00	4.52	26	0.14	11.67	1.00
3B	911	92.2	0.10	100.00	4.28	9	0.2	9.8	0.94
3D	112	70.15	0.63	98.20	8.49	5	0.38	17.22	1.00
4A	513	103.02	0.20	99.41	10.14	0	0.04	0.51	1.00
4B	360	78.41	0.22	99.72	6.52	17	0.32	10.59	0.87
4D	228	77.46	0.34	98.24	6.46	3	0.24	9.43	0.98
5A	312	114.24	0.37	99.04	6.77	15	0.36	12.07	0.99
5B	417	102.51	0.25	99.04	6.99	24	0.39	13.85	0.98
5D	195	96.47	0.50	97.94	8.92	1	0.32	10.62	0.99
6A	277	68.17	0.25	100.00	4.97	0	0.21	9.17	0.96
6B	110	38.94	0.36	100.00	3.58	1	1.36	18.19	0.99
6D	243	55.73	0.23	99.59	5.02	13	0.32	9.44	0.86
7A	578	88.21	0.15	100.00	4.40	0	0.22	11.94	0.99
7B	664	89.51	0.14	99.70	9.39	34	0.2	9.49	0.99
7D	177	121.37	0.69	97.73	7.67	17	0.33	11.81	1.00
Total	8751	1685.32	—	—	—	194	—	—	—
Average	—	—	0.19	99.26	—	—	—	—	—

Chromosome ID, linkage group number; SLAF number, the number indicates the total number of markers on a linkage group; Total distance, the total genetic distance of markers on a linkage group; Average distance Average genetic distance among all markers of linkage group; Max gap, the largest gap in the linkage group. The smaller the maximum gap, the more uniform the map is; Gaps < = 5 (%), the proportion of gap length less than 5 cM in the linkage group. The higher the proportion, the more uniform the representative map. BS, the number of partial segregation markers. SP (%), Singleton percent, the proportion of double exchange sites. Miss, missing proportion or deletion rate. Spearman: Spearman rank correlation coefficient. The closer the coefficient is to 1, the better the collinearity.

### Correlation of WUE with other traits

**Figure 2:**
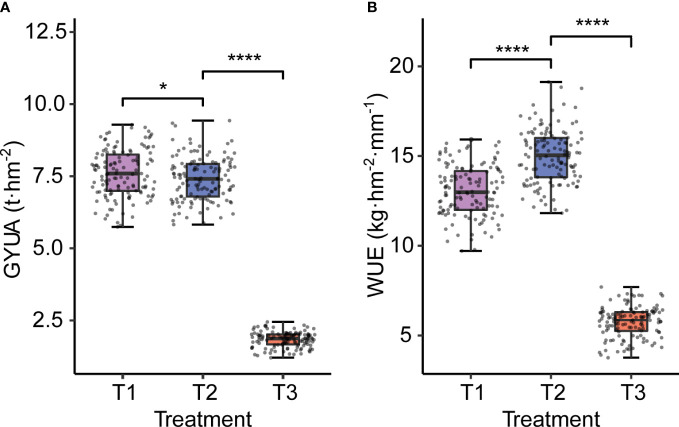
GYUA and WUE under different water treatments. **(A)** GYUA in different treatments. **(B)** WUE in different treatments. ****P < 0.0001, *P < = 0.05.

Water treatment affected both yield and WUE. With decreasing water treatments, yield decreased significantly, and the trend of GYUA was T1>T2>T3, while WUE showed a trend of first increasing and then decreasing, with high statistical significance. The tendency of WUE was T2> T1> T3 (**Figure 2**).

**Figure 3:**
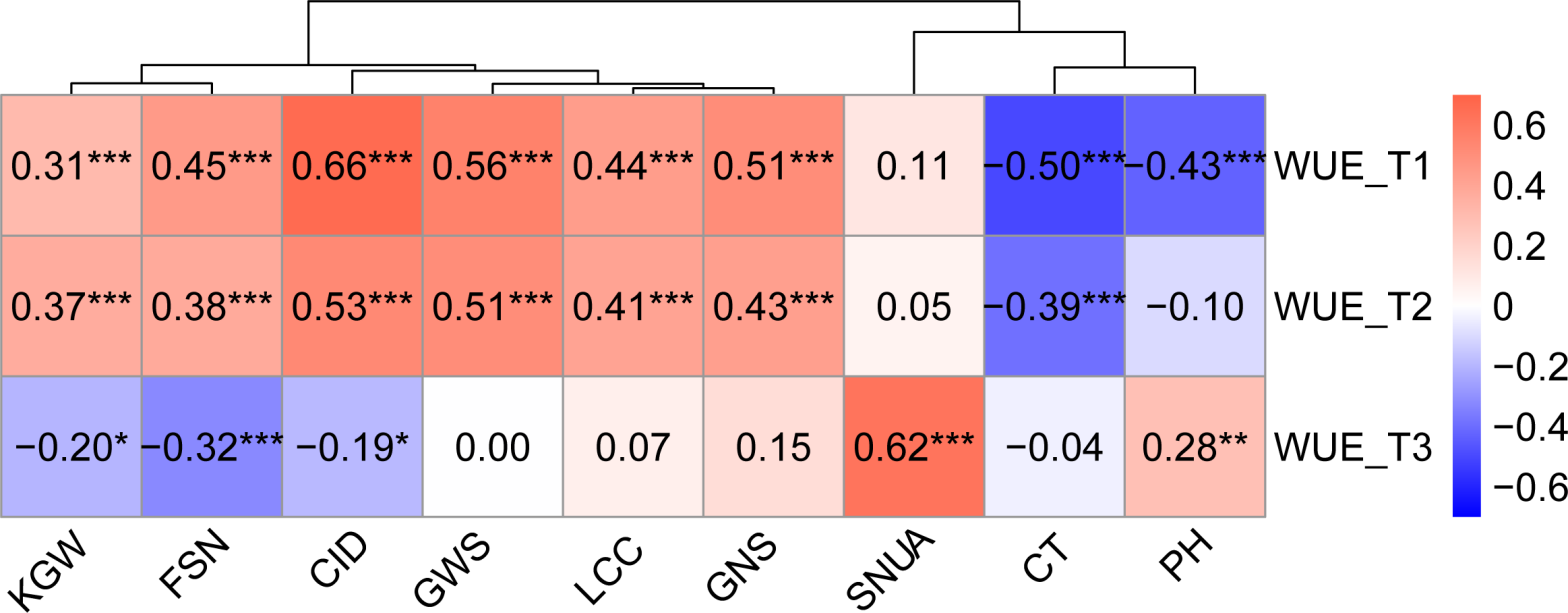
Correlation between WUE and other traits in different treatments. Pearson correlation coefficient in 0.6-0.8 is strong correlation, 0.4-0.6 is medium correlation, 0.2-0.4 is weak correlation, 0.0-0.2 is very weak correlation or no correlation. *** is *P*< 0.001, ** is *P*< 0.01, * is *P*< 0.05.

Correlation analysis illustrated that WUE was significantly correlated with other traits across treatments, and the degree of correlation varied with the environment. Except for KGW, SNUA and PH, the correlations between WUE and the other six traits were weakened with decreasing levels of irrigation, especially in the T3 treatment, which not only further weakened the correlations, but also changed the direction of some correlations (**Figure 3**). Specifically, under the T1 treatment, WUE was significantly positively correlated with KGW, FSN, CID, SWPS, LCC, GNS, and showed a strongly significant negative correlation with CT and PH. Under the T2 treatment the correlation between WUE and various traits were very similar to T1, except for a lack of correlation with PH.Under the T3 treatment, WUE was positively correlated with SNUA and PH, negatively correlated with FSN (*P* < 0.001), and negatively correlated with KGW and CID (**Figure 3**).

### QTL mapping of WUE and related traits

**Figure 4:**
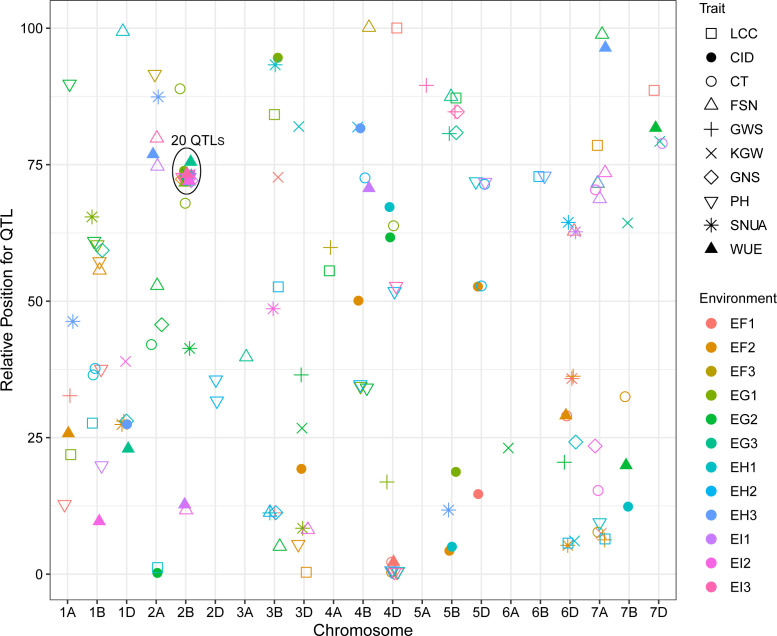
Distribution of QTLs for WUE and related traits across wheat chromosomes. Relative position is the percentage of genetic distance of trait location of the total genetic distance of chromosome. In legend, F, G, H and I represent the years 2017, 2018, 2019 and 2020 respectively, and 1, 2 and 3 represent T1, T2 and T3 water treatments, respectively. The ellipse plots the major QTLs (n=20) of WUE and related traits.

QTL mapping showed that the sites controlling WUE were distributed on 1A, 1B, 1D, 2A, 2B, 4B, 4D, 6D, 7A, 7B and 7D. Among these, the main effect locus was *QWue.acn-2B* (2B.2, 2B.3 and 2B.4; LOD 4.7-8.4), which was detected under different treatments in multiple years. The size of the QTL interval was ~2 cM and the phenotypic contribution rate was 15.6%-26.3%. The direction of the effect for WUE was environment specific for this locus: under T1 and T2 treatments Ningchun 27 had a positive effect, while under the T3 treatment the Ningchun 4 allele performed better. Chromosome 2B also harbored QTLs for KGW, GWS, GNS, PH, CID, LCC, and CT, which were mostly overlapping with *QWue.acn-2B*, and each of these has a fixed additive effect direction. The superior alleles for *QLcc-2B*, *QCt-2B*, and the inferior allele *qPH-2B* all came from Ningchun 27 (**Figure 4**, **Table 2**).

**Table 2:** Main effect loci for WUE and related traits.

QTL		Env.	Pos. (cM)	Left Marker	Right Marker	Left CI	Right CI	LOD	PVE (%)	Add.
*QLcc.acn-2B*		EI2	92.1	*Marker352143*	*Marker762683*	92.0	92.4	5.5	21.5	1.4
*QCid.acn-2B.1*		EG2	91.0	*Marker650772*	*Marker580039*	90.5	91.5	7.7	27.1	0.5
*QCid.acn-2B.2*		EF1	91.6	*Marker650772*	*Marker580039*	90.4	91.6	9.9	24.8	0.4
*QCid.acn-2B.3*		EG3	92.1	*Marker352143*	*Marker762683*	92.0	92.3	8.2	27.2	0.3
*QCid.acn-2B.4*		EG1	93.3	*Marker1025623*	*Marker1330209*	93.0	94.0	8.8	18.0	0.3
*QGws.acn-2B*		EG3	92.1	*Marker352143*	*Marker762683*	92.0	92.4	5.4	17.0	0.1
*QGws.acn-2B*		EI2	92.1	*Marker352143*	*Marker762683*	92.0	92.4	9.2	28.5	0.2
*QKgw.acn-2B*		EG2	92.1	*Marker352143*	*Marker762683*	92.0	92.3	14.8	19.6	4.0
*QKgw.acn-2B*		EI2	92.1	*Marker352143*	*Marker762683*	92.0	92.4	4.7	16.8	1.9
*QGns.acn-2B*		EF2	91.0	*Marker650772*	*Marker580039*	90.5	91.5	6.3	20.9	2.5
*QGns.acn-2B*		EI1	91.0	*Marker650772*	*Marker580039*	90.5	91.5	6.0	15.7	2.8
*QPh.acn-2B.1*		EH1	91.3	*Marker650772*	*Marker580039*	90.4	91.6	5.9	13.5	-4.3
*QPh.acn-2B.2*		EG3	91.6	*Marker650772*	*Marker580039*	90.5	91.6	6.9	20.9	-4.6
*QPh.acn-2B.2*		EH3	91.6	*Marker650772*	*Marker580039*	90.6	91.6	5.8	16.0	-4.1
*QPh.acn-2B.2*		EI3	91.6	*Marker650772*	*Marker580039*	90.7	91.6	12.0	12.2	-4.8
*QWue.acn-2B.2*		EG1	90.5	*Marker650772*	*Marker580039*	90.4	91.6	4.7	15.6	0.9
*QWue.acn-2B.2*		EH1	90.5	*Marker650772*	*Marker580039*	90.4	91.6	7.0	22.8	0.8
*QWue.acn-2B.3*		EI2	91.0	*Marker650772*	*Marker580039*	90.5	91.5	4.0	26.3	1.5
*QWue.acn-2B.4*		EI3	92.1	*Marker352143*	*Marker762683*	92.0	92.4	8.4	26.1	-0.8
*QWue.acn-2B.5*		EG3	95.3	*Marker1025623*	*Marker1330209*	94.1	96.2	5.0	16.0	-0.7

LeftCI and Right CI: Confidence interval calculated by one-LOD drop from the estimated QTL position. F, G, H and I represent the years 2017, 2018, 2019 and 2020 respectively, and 1, 2 and 3 represent T1, T2 and T3 water treatments, respectively. Env. stands for environment. Add. indicates additive effect. PVE the percentage of phenotypic variation explanations.

**Figure 5:**
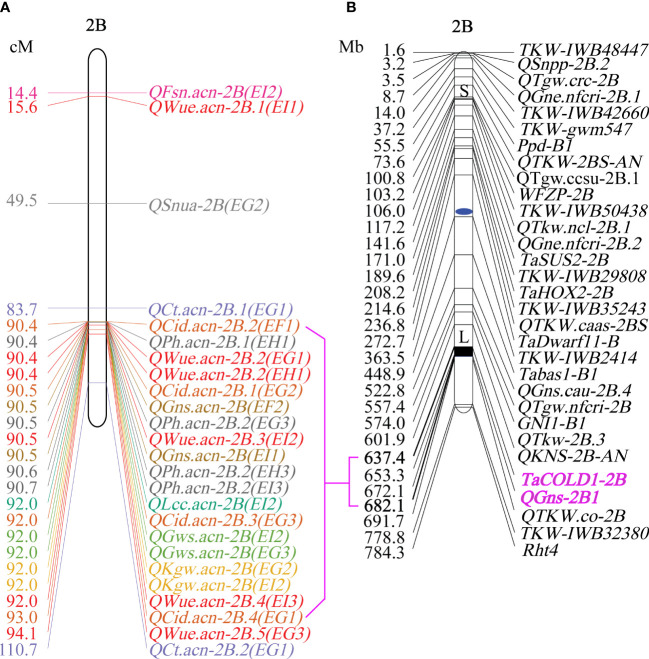
The map of WUE and related traits on chromosome 2B. **(A)** Genetic position of WUE and related traits on chromosome 2B. **(B)** Physical map of yield component traits on chromosome 2B (Dong et al., 2019; Cao et al., 2020).

The major QTL *QFsn.acn-7A* (*7A.1*, *7A.2*, *7A.3*, and *7A.4*) controlling spikelet number was detected across multiple treatments and years, with a genetic interval of 5 cM, with PVE of 13.0-20.8%. The *QCt.acn-1B.1* and *QCt.acn-1B.2* were detected at similar positions, and *QCt.acn-1B.2* explained more than 20% of canopy temperature variation. The *QCid.acn-4D.1* was close to *QCT.acn-4D.2*, but their additive effects were in the opposite direction. The *QCid.acn-5D.1* and *QCid.acn-5D.2* were adjacent, but their additive effects were in the opposite direction.The *QGws-3B* was detected in the same water treatment in two years, which explained more than 20% of the grain weight per ear variation. In addition, stable QTL that control plant height were also detected on 1B, 4B, 2D, 4D, and 5D. Among these, the *QPh.acn-2D* interval contains the Reduced Height (*Rht*) dwarfing gene, and *QPh.acn-4D* is adjacent to *TaTD1*. Outside of chromosome 2B, with the exception of *QPh.acn-4D.1*, *QCid.acn-4D.2* and *QWue.acn-4D* (EF1) which are similar in position, no co-localization of WUE QTL with those of related traits was found. For the locus, *QWue.acn-4D* (LOD of 3.8) with a PVE of 11.6%, the allele for increased WUE, decreased canopy temperature, and decreased plant height was from Ningchun 4 (**Figure 5**, **Table 2**, **Supplementary Table S4**).

### Effects of different alleles of *Qwue.acn-2B* on WUE and related traits

**Figure 6:**
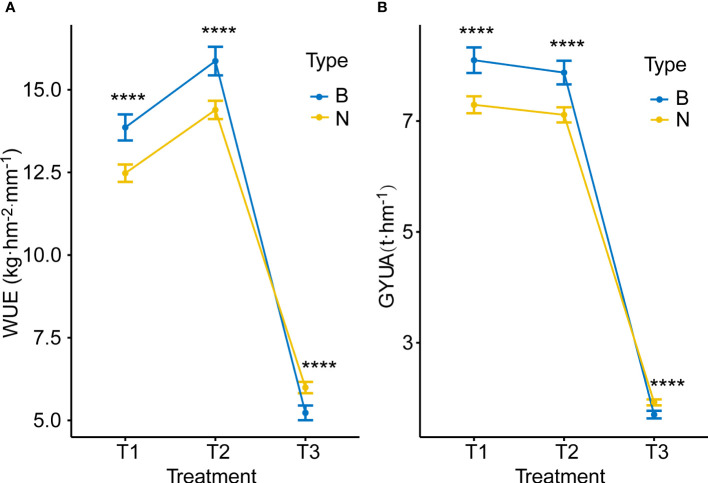
Allele effects for WUE and GYUA under different treatments **(A)** WUE of type B (*QWue.acn-2B* ^Ningchun 27^) and type N (*QWue.acn-2B* ^Ningchun 4^) lines under different treatments. **(B)** GYUA of type B and type N lines under different treatments. **** *P* < 0.0001.

**Figure 7:**
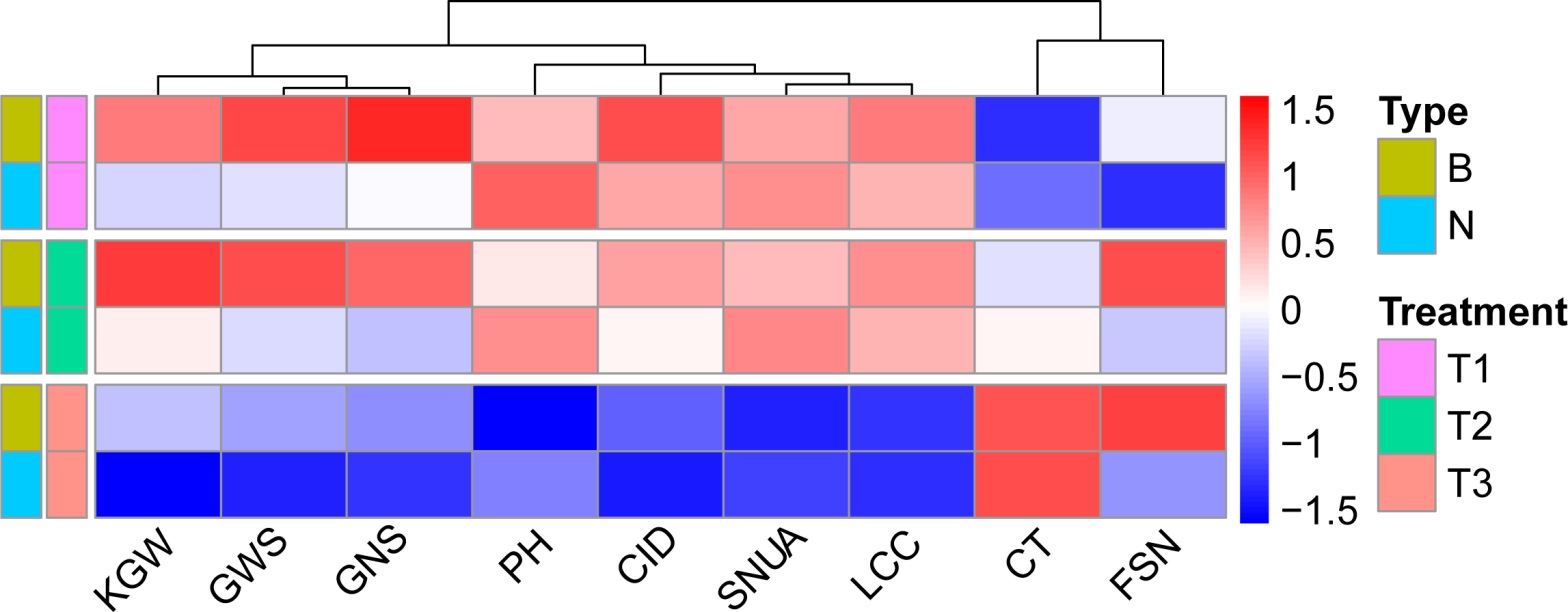
The performance of WUE related traits in different *QWue.acn-2B* genotypes.

We then grouped RILs as type B, having Ningchun 27 allele at *QWue.acn-2B* (*QWue.acn-2B* ^Ningchun 27^), or type N, having the Ningchun 4 allele (*QWue.acn-2B* ^Ningchun 4^), according to the flanking markers. Overall, the two groups responded consistently across environments for both WUE, T2 > T1 > T3, and yield, T1 > T2 > T3 (**Figure 6**). The effect of *QWue.acn-2B* was consistent across T1 and T2 environments, with type B being associated with significantly higher yield than type N, but this relationship was opposite in the T3 treatment (**Figure 6**). The performances of the other 9 traits (KGW, GWS, GNS, PH, CID, SNUA, LCC, CT and FSN) related to *QWue.acn-2B* under different treatments were compared, and the direction of the additive effect was consistent with that of parental phenotypic difference. In specific analysis, compared with type N, type B had higher grain CID, KGW, GWS, more GNS and FSN, and lower PH across treatments. Under the T2 treatment, type B LCC was higher than that of type N, CT was the opposite, whereas LCC or CT was little changed between types under T3 treatment (**Figure 7**).

The differences in yield and WUE were finally reflected in the difference in the dominant components of yield composition under the same water conditions. The analysis of the yield components of genotypes with different WUE shows that under T1 and T2 treatments, KGW and GWS are the main factors influencing WUE. Genotypes with higher KGW and GWS tend to have higher WUE, and under T3 treatment, SNUA is the main factor influencing WUE, and genotypes with fewer ears tend to have lower WUE (**Table 3**). Therefore, without water stress before flowering (T1&T2), type B with high grain weight was more dominant than type N in WUE and yield, but under rain-fed conditions (T3) type B had less harvest spikes and had lower WUE and yield.

**Table 3:** Correlation of WUE related traits in different *QWue.acn-2B* genotypes.

QWue.acn-2B	Treatment	KGW	SNUA	GNS	GWS
N (n=79)	T1	0.14	0.30**	0.32**	0.34**
	T2	0.17	0.28*	0.26*	0.29**
	T3	-0.11	0.52***	0.15	0.09
B (n=49)	T1	0.27	0.15	0.46***	0.53***
	T2	0.31*	0.12	0.29*	0.41*
	T3	-0.11	0.62***	0.25	0.16

*** P < 0.001, ** is P < 0.01, * is P < 0.05.

## Discussion

In this study, the loci controlling WUE and its main related traits were found to be scattered across the 21 wheat chromosomes. Most loci were environment-specific, and only a few loci were stable across environments, suggesting the quantitative inheritance of WUE and its related traits and the complexity of regulation of trait formation. *QWue.acn-2B* and some sites controlling CID, grain weight per ear, chlorophyll content, 1000 grain weight and plant height mapped to the same or similar positions to form gene clusters, showing pleiotropic effect, demonstrating that t high WUE materials can at the same time increase yield. The *QWue.acn-2B* locus has different effects on maintaining or increasing WUE or yield under different water use conditions. In the Yellow River irrigation area of Ningxia, the water-saving and yield increasing effect of families of the *QWue.acn-2B* ^Ningchun 27^ allele is stronger than that of families with *QWue.acn-2B* ^Ningchun 4^. In the rain fed area of Southern Ningxia, *QWue.acn-2B* ^Ningchun 27^ no longer contributes to water-saving and yield, and even reduces WUE. These opposing effects need to be further dissected through examining the trait changes closely related to WUE.

In the process of modern variety breeding, the number of grains per ear and 1000 grain weight often show a negative correlation. Finding the balance between the two is often the key to increasing yield. In the past 10 years (up to 2020), 205 QTLs related to yield component traits of wheat have been found in a multi repeat environments, most of which are QTLs for 1000 grain weight and grain number per ear. Through homology comparison, 33 and 9 yield component genes confirmed to be functional in wheat have been isolated from rice and wheat related species, respectively (Cao et al., 2020), but few genes/QTLs have positive effects on two or more yield components at the same time. In our study, *QKgw.acn-2B* and *QGns.acn-2B* loci overlapped, and the phenotypic variance explained was more than 15%. The direction of additive effect was the same, showing the coordination and unity of grain number per ear and 1000 grain weight, which promoted increased yield and high WUE under favorable conditions and showed great application potential and value.

The SPAD value of wheat varieties is positively correlated with the total nitrogen content in leaves (Zhu et al., 2005), which can accurately reflect the transpiration efficiency (TE) under drought conditions (Fotovat et al., 2007). Major SPAD QTLs were found in 2B and 3B of the durum wheat dihaploid population, with a contribution rate of about 14% (Knox, 2013). QTLs controlling SPAD were detected on chromosomes 6A, 7A, 1B and 1D of common wheat, with a contribution rate of 11.4-30.8% (Talukder et al., 2014). SPAD QTLs play an important role in drought resistance and heat tolerance. Lower leaf temperature is related to the adaptability of stomatal conductance and higher photosynthetic rate (Lu et al., 1998; Siddique et al., 2000). The colder canopy distributes assimilates to deeper roots to adapt to drought conditions and increase yield (Lopes and Reynolds, 2010). It is reported that the air crown temperature difference (CTD) and economic yield are similar to each other in 3BL and 5DL, which produce lower crop canopy temperature and higher economic yields (Mason et al., 2013). Consistent with the previous research results, this study found that the locus controlling SPAD/LCC and CT on chromosome 2B overlaps or is adjacent to *QWue.acn-2B*. The favorable allele has the effect of increasing chlorophyll content and reducing canopy temperature, and can promote the formation of high WUE traits.

Plant height has an important impact on yield components. Several studies have implicated *Rht* in yield (Tshikunde et al., 2019; Gao et al., 2020; Wen et al., 2022). For example, plant yield was increased by 16.4 and 8.2% in the *Rht12* dwarf lines (Chen et al., 2018), *Rht25b* showed significant pleiotropic effects on yield components (Mo et al., 2018), *Rht4* and *Rht8* could reduce plant height to a desirable level and improve yield related traits in the rainfed environment (Du et al., 2018). It was shown that *Rht-B1b*(4BS) + *Rht4*(2BL) gene can increase the number of grains per ear and reduce 1000 grain weight (Liu et al., 2017). Similarly, was it was shown that *Rht14* can reduce 1000 grain weight (Duan et al., 2020), and *Rht24*(6A) gene can increase 1000 grain weight (Tian et al., 2017). In this study, *QPh.acn-2B* overlaps *QWue.acn-2B*, but is not in the same position as *Rht-B1b*, *Rht4*, and *Rht24*. Although *TaCOLD1-2B* and *QGns-2B1* are included in the *QPh.acn-2B* region, they are not related to *QPh.acn-2B* in this study, so it may be a new dwarf gene. *QWue.acn-2B* leads to higher grain number per ear, 1000 grain weight and grain weight per ear. We speculate that *QPh.acn-2B* may also make an important contribution to the increase of grain number per ear, 1000 grain weight and grain weight per ear, which needs to be further verified.

CID (Δ) is often used to indicate WUE and yield status of crops in arid and semi-arid areas. Many field experiments show that it is usually positively correlated with grain yield under both drought and sufficient water conditions (Condon et al., 1987; Craufurd et al., 1991; Morgan et al., 1993; Sayre et al., 1995). Especially when the soil water supply is sufficient in the early stage of growth, CID has a positive correlation with aboveground dry matter. When the soil is seriously dry in the late stage, the wheat yield of low CID genotype is often higher (Condon et al., 1993). Higher grain CID genotypes tend to have higher harvest index (HI), reflecting that grain filling is more dependent on pre anthesis dry matter reserves, which are obtained when the early growth of plants is not under water stress (Condon et al., 2004). This study found that under the condition of good soil water supply before anthesis (T1), grain CID was significantly positively correlated with yield. This relationship was maintained even if the soil water decreased after anthesis (T2), while under rain fed conditions (T3), grain CID was significantly negatively correlated with yield, consistent with previous findings. This can be explained by the fact that *QCid.acn-2B* and *QWue.acn-2B* are at the same or similar position, and the direction of their additive effects is the same under T1&T2, but opposite under T3. High CID is related to high CO_2_ concentration in the intercellular space which is caused by high stomatal conductance (Farquhar and Richards, 1984). When soil water content is very limited, the high-yield genotype (high CID) will be at a disadvantage due to its high stomatal conductance. In arid environments with deep soil water supplies, the high-yield genotype (high CID) will show the greatest advantage (Blum, 1993; Blum, 1996).

In this study, the economic yield or WUE of type B carrying many good traits in different environments (T1&T2 vs T3) were quite the opposite. This is because under the condition of good soil water supply before flowering, ear grain weight and 1000 grain weight are the main factors determining the height of WUE. The high CID genotype (B) tends to have higher WUE and yield because of its better filling characteristics (higher 1000 grain weight and ear grain weight). The number of ears harvested under rain-fed conditions is the main factor affecting the degree of WUE, and the high CID genotype will produce less ears. The reason why the number of harvested spikes is less may be that high CID induces greater stomatal conductance and transpiration dissipation, which intensifies the competition for limited soil water among individuals and makes it difficult for those individuals to form spikes in this adverse microenvironment. Based on the above analysis, for areas with good soil water storage or supply before flowering, the selection of characters for higher grain CID, 1000 grain weight and ear grain weight is helpful to improve economic yield and WUE. In rain fed areas, especially in areas with very limited soil water before flowering, the selection of characters for lower grain CID and more harvested ears is helpful to increase the economic yield and WUE. In conclusion, type B is suitable for use in irrigation areas, and type N is suitable for use in rain-fed areas.

Although *QWue.acn-2B* was identified as the main effect loci in the initial mapping, the physical interval is still large. In the future, it is necessary to construct a secondary segregating population for fine mapping. In addition, as multiple traits are located at similar or overlapping positions, it is also necessary to decompose the genetic effects based on the further segregation of traits to clarify the relationship between them. As *QWue.acn-2B* itself comes from the main varieties and is closely linked with many favorable traits, its flanking molecular markers can be used to introduce excellent chromosome fragments into other materials through molecular marker assisted technology in the future. This effort will verify the effectiveness of *QWue.acn-2B* and speed up the cultivation of high-yield, high-efficiency, and water-saving varieties.

## Data availability statement

The datasets presented in this study can be found in online repositories. The names of the repository/repositories and accession number(s) can be found in the article/**Supplementary Material**.

## Author contributions

SL conceived the project and constructed the recombinant inbred line population (Ningchun 4 x Ningchun 27). HB, XL, SM, and XC performed field tests. JH mapped the traits, analyzed the data and wrote the manuscript. All authors contributed to the article and approved the submitted version.

## References

[B1] Acevedo E. (1993). “Potential of carbon isotope discrimination as a selection criterion in barley breeding,” in Stable isotopes and plant carbon-water relations. Eds. Ehleringer J. R. Hall A. E. Farquhar G. D. (New York: Academic Press), 399–417.

[B2] Acevedo M. Zurn J. D. Molero G. Singh P. He X. Aoun M.. (2018). “The role of wheat in global food security,” in Agricultural development and sustainable intensification. Ed. Nagothu U. S. (London, UK: Routledge), 81–110.

[B3] Amani I. Fischer R. A. Reynolds M. P. (2010). Canopy temperature depression association with yield of irrigated spring wheat cultivars in a hot climate. J. Agron. Crop Sci. 176, 119–129. doi: 10.1111/j.1439-037x.1996.tb00454.x

[B4] Balota M. Sowinska M. Buschmann C. Lichtenthaler H. K. Babani F. (1999). Fluorescence techniques as suitable methods to discriminate wheat genotypes under drought and high-temperature conditions. Proc. SPIE Int. Soc Opt. Eng. Proc. 3707, 103–113. doi: 10.1117/12.351334

[B5] Blum A. (1993). “Selection for sustained production in water-deficit environments,” in International crop science. I. Eds. Buxton D. R. Forsberg R. A. Blad B. L. Asay K. H. Paulsen G. M. Wilson R. F., CSSA, Madison. WI, 343–347.

[B6] Blum A. (1996). “Yield potential and drought resistance: are they mutually exclusive?,” in Yield potential in wheat: breaking the barriers. Eds. Reynolds M. P. Rajaram S. McNab A. (Mexico, D. F: CIMMYT), 76–89.

[B7] Cao S. Xu D. Hanif M. Xia X. He Z. (2020). Genetic architecture underpinning yield component traits in wheat. Theor. Appl. Genet. 133, 1811–1823. doi: 10.1007/s00122-020-03562-8 32062676

[B8] Chao J. Li Z. Sun Y. Aluko O. O. Wu X. Wang Q.. (2021). MG2C: a user-friendly online tool for drawing genetic maps. Mol. Hortic. 1, 16. doi: 10.1186/s43897-021-00020-x PMC10514940 37789491

[B9] Chen L. Du Y. Lu Q. Chen H. Meng R. Cui C.. (2018). The photoperiod-insensitive allele ppd-D1a promotes earlier flowering in Rht12 dwarf plants of bread wheat. Front. Plant Sci. 9. doi: 10.3389/fpls.2018.01312 PMC6204387 30405643

[B10] Condon A. G. Farquhar G. D. Richards R. A. (1990). Genotypic variation in carbon isotope discrimination and transpiration efficiency in wheat-leaf gas exchange and whole plant studies. Aust. J. Plant Physiol. 17, 9–22. doi: 10.1071/PP9900009

[B11] Condon A. G. Richards R. A. Farquhar G. D. (1987). Carbon isotope discrimination is positively correlated with grain yield and dry matter production in field-grown wheat. Crop Sci. 27, 996–1001. doi: 10.2135/CROPSCI1987.0011183X002700050035X

[B12] Condon A. G. Richards R. A. Farquhar G. D. (1993). Relationships between carbon isotope discrimination, water use efficiency and transpiration efficiency for dryland wheat. Crop Pasture Sci. 44, 1693–1711. doi: 10.1071/AR9931693

[B13] Condon A. G. Richards R. A. Rebetzke G. J. Farquhar G. D. (2002). Improving intrinsic water-use efficiency and crop yield. Crop Sci. 42, 122–131. doi: 10.2135/cropsci2002.1220 11756262

[B14] Condon A. G. Richards R. A. Rebetzke G. J. Farquhar G. D. (2004). Breeding for high water-use efficiency. J. Exp. Bot. 55, 2447–2460. doi: 10.1093/jxb/erh277 15475373

[B15] Craufurd P. Q. Austin R. B. Acevedo E. Hall M. A. (1991). Carbon isotope discrimination and grain yield in barley. Field Crops Res. 27, 301–313. doi: 10.1016/0378-4290(91)90038-W

[B16] Dong H. Yan S. Liu J. Liu P. Sun J. (2019). TaCOLD1 defines a new regulator of plant height in bread wheat. Plant Biotechnol. J. 17, 687–699. doi: 10.1111/pbi.13008 30183124 PMC6381784

[B17] Duan S. Zhao Z. C. Qiao Y. Cui C. G. Morgunov A. Condon A. G.. (2020). GAR dwarf gene Rht14 reduced plant height and affected agronomic traits in durum wheat (Triticum durum). Field Crops Res. 248, 107721. doi: 10.1016/j.fcr.2020.107721

[B18] Du Y. Chen L. Wang Y. Yang Z. Saeed I. Daoura B. D.. (2018). The combination of dwarfing genes Rht4 and Rht8 reduced plant height, improved yield traits of rainfed bread wheat (Triticum aestivum L.). Field Crops Res. 215, 149–155. doi: 10.1016/j.fcr.2017.10.015

[B19] Ehdaie B. Waines J. G. (1993). Variation in water-use efficiency and its components in wheat: I. well-watered pot experiment. Crop Sci. 33, 294–299. doi: 10.2135/cropsci1993.0011183X003300020016x

[B20] Ehdaie B. Waines J. G. (1994). Growth and transpiration efficiency of near-isogenic lines for height in a spring wheat. Crop Sci. 34, 1443–1451. doi: 10.2135/cropsci1994.0011183X003400060004x

[B21] Farquhar G. D. Ehleringer J. R. Hubick K. T. (1989). Carbon isotope discrimination and photosynthesis. Annu. Rev. Plant Physiol. Plant Mol. Biol. 40, 503–537. doi: 10.1146/annurev.pp.40.060189.002443

[B22] Farquhar G. D. Richards P. A. (1984). Isotopic composition of plant carbon correlates with water-use efficiency of wheat genotypes. Aust. J. Plant Physiol. 11, 539–552. doi: 10.1071/PP9840539

[B23] Fischer R. A. Turner N. C. (1978). Plant productivity in the arid and semiarid zones. Annu. Rev. Plant Biol. 29, 277. doi: 10.1146/annurev.pp.29.060178.001425

[B24] Fotovat R. Valizadeh M. Toorchi M. (2007). Association between water-use efficiency components and total chlorophyll content (SPAD) in wheat (Triticum aestivum L.) under well-watered and drought stress conditions. J. Food Agric. Environ. 5, 225–227. Available at: http://www.world-food.net/scientificjournal.php.

[B25] Franks P. J. Doheny-Adams T. Britton-Harper Z. J. Gray J. E. (2015). Increasing water-use efficiency directly through genetic manipulation of stomatal density. New Phytol. 207, 188–195. doi: 10.1111/nph.13347 25754246

[B26] Gago J. Douthe C. Florez-Sarasa I. Escalona J. M. Galmes J. Fernie A. R.. (2014). Opportunities for improving leaf water use efficiency under climate change conditions. Plant Sci. 226, 108–119. doi: 10.1016/j.plantsci.2014.04.007 25113456

[B27] Gao Z. Wang Y. Tian G. Zhao Y. Li C. Cao Q.. (2020). Plant height and its relationship with yield in wheat under different irrigation regime. Irrig. Sci. 38, 365–371. doi: 10.1007/s00271-020-00678-z

[B28] Gorny A. G. (1999). Effects of d-genome substitutions on the water use efficiency and of the langdon durum wheat to reduced nitrogen nutrition. Cereal Res. Commun. 27, 83–90. doi: 10.1007/BF03543923

[B29] Hall A. E. Mutters R. G. Farquhar G. D. (1992). Genotypic and drought-induced differences in carbon isotope discrimination and gas exchange of cowpea. Crop Sci. 32, 1–6. doi: 10.2135/cropsci1992.0011183X003200010002x

[B30] Hall A. E. Mutters R. Hubick K. T. Farquhar G. D. (1990). Genotypic differences in carbon isotope discrimination bycowpea under wet and dry field conditions. Crop Sci. 30, 300–305. doi: 10.2135/cropsci1990.0011183X003000020011x

[B31] Handley L. L. Nevo E. Raven J. A. Martínez-Carrasco R. Scrimgeour C. M. Pakniyat H.. (1994). Chromosome 4 controls potential water use efficiency (δ13C) in barley. J. Exp. Bot. 45, 1661–1663. doi: 10.1093/jxb/45.11.1661

[B32] Henry R. J. Furtado A. Rangan P. (2018). Wheat seed transcriptome reveals genes controlling key traits for human preference and crop adaptation. Curr. Opin. Plant Biol. 45, 231–236. doi: 10.1016/j.pbi.2018.05.002 29779965

[B33] Hsiao T. C. (2000). Sensitivity of growth of roots versus leaves to water stress: biophysical analysis and relation to water transport. J. Exp. Bot. 51, 1595–1616. doi: 10.1093/jexbot/51.350.1595 11006310

[B34] Hubick K. T. Farquhar G. D. (1989). Carbon isotope discrimination and the ratio of carbon gained to water lost in barley cultivars. Plant Cell Environ. 12, 795–804. doi: 10.1111/j.1365-3040.1989.tb01641.x

[B35] Hubick K. T. Farquhar G. D. Shorter R. (1986). Correlation between water-use efficiency and carbon isotope discrimination in diverse peanut (Arachis) germplasm. Aust. J. Plant Physiol. 13, 803–816. doi: 10.1071/PP9860803

[B36] Hu M. J. Zhang H. P. Liu K. Cao J. J. Wang S. X. Hao J.. (2016). Cloning and characterization of TaTGW-7A gene associated with grain weight in wheat *via* SLAF-seq-BSA. Front. Plant Sci. 7. doi: 10.3389/fpls.2016.01902 PMC5167734 28066462

[B37] Islam M. R. Haque K. Akter N. Karim M. A. (2014). Leaf chlorophyll dynamics in wheat based on SPAD meter reading and its relationship with grain yield. Sci. Agric. 8, 13–18. doi: 10.15192/PSCP.SA.2014.4.1.1318

[B38] Ismail A. M. Hall A. E. (1992). Correlation between water-use efficiency and carbon isotope discrimination in diverse cowpea genotypes and isogenic lines. Crop Sci. 32, 7–12. doi: 10.2135/cropsci1992.0011183X003200010003x

[B39] Khazaei H. Mohammady S. (2010). Relationships between carbon isotope discrimination(Ä), grain yield and dry matter production in durum wheat. Pajouhesh. Sazandegi 86, 20–27. Available at: https://agris.fao.org/agris-search/search.do?recordID=IR2011005190.

[B40] Khler I. H. Macdonald A. J. Schnyder H. (2015). Last-century increases in intrinsic water-use efficiency of grassland communities have occurred over a wide range of vegetation composition, nutrient inputs, and soil pH. Plant Physiol. 170, 881–890. doi: 10.1104/pp.15.01472 26620525 PMC4734565

[B41] Knox R. E. (2013). “Quantitative trait loci for chlorophyll content in durum wheat,” at the XXI International Conference on Plant & animal genome: San Diego, CA. Available at: https://pag.confex.com/pag/xxi/webprogram/Paper7122.html.

[B42] Kole C. Muthamilarasan M. Henry R. Edwards D. Sharma R. Abberton M.. (2015). Application of genomics-assisted breeding for generation of climate resilient crops: progress and prospects. Front. Plant Sci. 6. doi: 10.3389/fpls.2015.00563 PMC4531421 26322050

[B43] Kuneg A. Naz A. Dedeck O. (2007). AB-QTL analysis in winter wheat: I. synthetic hexaploid wheat ( t.turgidum ssp.dicoccoides × t.tauschii ) as a source of favorable alleles for milling and baking quality traits. Theor. Appl. Genet. 115, 683–695. doi: 10.1007/s00122-007-0600-7 17634917

[B44] Lambrides C. J. Chapman S. C. Shorter R. (2004). Genetic variation for carbon isotope discrimination in sunflower: Association with transpiration efficiency and evidence for cytoplasmic inheritance. Crop Sci. 44, 1642–1653. doi: 10.2135/cropsci2004.1642

[B45] Li R. Li Y. Kristiansen K. Wang J. (2008). Soap: short oligonucleotide alignmentprogram. Bioinformatics 24, 713–714. doi: 10.1093/bioinformatics/btn025 18227114

[B46] Li X. J. Pan Z. D. (2005). A study on the grain-filling characteristic of different weight wheat. Rev. China Agric. Sci. Technol. 7, 26–30. Available at: http://en.cnki.com.cn/Article_en/CJFDTotal-NKDB200501007.htm.

[B47] Liu Y. X. Zhang J. L. Hu Y. G. Chen J. L. (2017). Dwarfing genes Rht4 and rht-B1b affect plant height and key agronomic traits in common wheat under two water regimes. Field Crop Res. 204, 242–248. doi: 10.1016/j.fcr.2017.01.020

[B48] Lopes M. S. Reynolds M. P. (2010). Partitioning of assimilates to deeper roots is associated with cooler canopies and increased yield under drought in wheat. Funct. Plant Biol. 37, 147–156. doi: 10.1071/FP09121

[B49] Lu Z. Percy R. G. Qualset C. O. Eduardo Z. (1998). Stomatal conductance predicts yields in irrigated pima cotton and bread wheat grown at high temperatures. J. Exp. Bot., 49, 453–460. doi: 10.1093/jxb/49.Special_Issue.453

[B50] Masle J. Gilmore S. R. Farquhar G. D. (2005). The ERECTA gene regulates plant transpiration efficiency in arabidopsis. Nature 10, 1–5. doi: 10.1038/nature03835 16007076

[B51] Mason R. E. Hays D. B. Mondal S. Ibrahim A. Basnet B. R. (2013). QTL for yield, yield components and canopy temperature depression in wheat under late sown field conditions. Euphytica, 194, 243–259. doi: 10.1007/s10681-013-0951-x

[B52] Mcintosh R. A. Yamazaki Y. Y. Dubcovsky J. Rogers J. Morris C. Appels R.. (2013). “Catalogue of gene symbols for wheat,” Agronomy Journal. (Yokohama, Japan: 12th international wheat genetics symposium).

[B53] Merah O. Deléens. E. Monneveux P. (2001). Relationships between carbon isotope discrimination, dry matter production, and harvest index in durum wheat. J. Plant Physiol. 158, 723–729. doi: 10.1078/0176-1617-00273

[B54] Monneveux P. Reynolds M. P. Trethowan R. Gonzalez-Santoyo H. Pena R. J. Zapata F. (2005). Relationship between grain yield and carbon isotope discrimination in bread wheat under four water regimes. Eur. J. Agron. 22, 231–242. doi: 10.1016/j.eja.2004.03.001

[B55] Morgan J. A. LeCain D. R. McCaig T. N. Quick J. S. (1993). Gas exchange, carbon isotope discrimination and productivity in winter wheat. Crop Sci. 33, 178–186. doi: 10.2135/cropsci1993.0011183X003300010032x

[B56] Mo Y. Vanzetti L. S. Hale I. Spagnolo E. J. Guidobaldi F. Al-Oboudi J.. (2018). Identification and characterization of Rht25, a locus on chromosome arm 6AS affecting wheat plant height, heading time, and spike development. Theor. Appl. Genet. 131, 2021–2035. doi: 10.1007/s00122-018-3130-6 29959472

[B57] Narasimhamoorthy B. Gill B. S. Fritz A. K. (2006). Advanced backcross QTL analysis of a hard winter wheat × synthetic wheat population. Theor. Appl. Genet. 112, 787–796. doi: 10.1007/s00122-005-0159-0 16463062

[B58] Pinto R. S. Reynolds M. P. Mathews K. L. McIntyre C. L. Olivares-Villegas J. J. Chap-man S. C. (2010). Heat and drought adaptive QTL in a wheat population designed to minimize con-founding agronomic effects. Theor. Appl. Genet. 121, 1001–1021. doi: 10.1007/s00122-010-1351-4 20523964 PMC2938441

[B59] Rebetzke G. J. Condon A. G. Richards R. A. Farquhar G. D. (2002). Selection for reduced carbon isotope discrimination increases aerial biomass and grain yield of rainfed bread wheat. Crop Sci. 42, 739–745. doi: 10.2135/cropsci2002.7390

[B60] Rebetzke G. J. Rattey A. R. Farquhar G. D. Richards R. A. Condon A. G. (2013). Genomic regions for canopy temperature and their genetic association with stomatal conductance and grain yield in wheat. Funct. Plant Biol. 40, 14–33. doi: 10.1071/FP12184 32481083

[B61] Richards R. A. (1992). The effect of dwarfing genes in spring wheat in dry environments. II. growth, water use and water-use efficiency. Aust. J. Agr. Res. 43, 529–539. doi: 10.1071/AR9920529

[B62] Rytter R. M. (2005). Water use efficiency, carbon isotope discrimination and biomass production of two sugar beet varieties under well-watered and dry conditions. J. Agron. Crop Sci. 191, 426–438. doi: 10.1111/j.1439-037X.2005.00162.x

[B63] Sayre K. D. Acevedo E. Austin. R. B. (1995). Carbon isotope discrimination and grain yield for three bread wheat germplasm groups grown at different levels of water stress. Field Crops Res. 41, 45–54. doi: 10.1016/0378-4290(94)00105-L

[B64] Siddique M. Hamid A. Islam M. S. (2000). Drought stress effects on water relation of wheat. Bot. Bull. Acad. Sinica. 41, 35–39. Available at: https://ejournal.sinica.edu.tw/bbas/content/2000/1/bot11-06.html.

[B65] Sivamani E. Bahieldin A. Wraith J. M. Al-Niemi T. Dyer W. E. Ho T.. (2000). Improved biomass productivity and water use efficiency under water deficit conditions in transgenic wheat constitutively expressing the barley HVA1 gene. Plant Sci. 155, 1–9. doi: 10.1016/s0168-9452(99)00247-2 10773334

[B66] Srivastava A. Srivastava P. Sharma A. Sarlach R. S. (2017). Canopy temperature an effective measure of drought stress tolerance in RIL population of wheat. Int. J. Plant Sci. 30, 59. doi: 10.5958/2229-4473.2017.00011.8

[B67] Sun X. Liu D. Zhang X. Li W. Liu H. Hong W.. (2013). SLAF-seq: an efficient method of large-scale *de novo* SNP discovery and genotyping using high-throughput sequencing. PloS One 8, e58700. doi: 10.1371/journal.pone.0058700 23527008 PMC3602454

[B68] Sun C. Zhang F. Yan X. Zhang X. Dong Z. Cui D.. (2017). Genome-wide association study for 13 agronomic traits reveals distribution of superior alleles in bread wheat from the yellow and huai valley of China. Plant Biotechnol. J. 15, 953–969. doi: 10.1111/pbi.12690 28055148 PMC5506658

[B69] Talukder S. K. Babar M. A. Vijayalakshmi K. Poland J. Prasad P. Bowden R.. (2014). Mapping QTL for the traits associated with heat tolerance in wheat (Triticum aestivum L.). BMC Genet. 15, 1–13. doi: 10.1371/journal.pone.0058700 25384418 PMC4234900

[B70] Tian X. L. Wen W. E. Xie L. Fu L. P. Xu D. A. Fu C.. (2017). Molecular mapping of reduced plant height gene Rht24 in bread wheat. Front. Plant Sci. 8, 1379. doi: 10.3389/fpls.2017.01379 28848582 PMC5550838

[B71] Tshikunde N. M. Mashilo J. Shimelis H. Odindo A. (2019). Agronomic and physiological traits, and associated quantitative trait loci (QTL) affecting yield response in wheat (Triticum aestivum L.): A review. Front. Plant Sci. 10. doi: 10.3389/fpls.2019.01428 PMC6848381 31749826

[B72] Wei Q. Wang W. Hu T. Hu H. Bao C. (2020). Construction of a SNP-based genetic map using SLAF-seq and QTL analysis of morphological traits in eggplant. Front. Genet. 11, 178. doi: 10.3389/fgene.2020.00178 32218801 PMC7078336

[B73] Wei Q. Wang Y. Qin X. Zhang Y. Zhang Z. Jing W.. (2014). An SNP-based saturated genetic map and QTL analysis of fruit-related traits in cucumber using specific-length amplified fragment (SLAF) sequencing. BMC Genomics 15, 1158. doi: 10.1186/1471-2164-15-1158 25534138 PMC4367881

[B74] Wen S. Zhang M. Tu K. Fan C. Tian S. Bi C.. (2022). A major quantitative trait loci cluster controlling three components of yield and plant height identified on chromosome 4B of common wheat. Front. Plant Sci. 12, 799520. doi: 10.3389/fpls.2021.799520 35087558 PMC8786729

[B75] Xing X. Yuan H. Li S. Trethowan R. Monneveux P. (2007). Relationship between carbon isotope discrimination and, grain yield in spring wheat cultivated under different water regimes. J. Integr. Plant Biol. 49, 1497–1507. doi: 10.1111/j.1672-9072.2007.00562.x

[B76] Yadav M. R. Choudhary M. Singh J. Lal M. K. Jha P. K. Udawat P. (2022). Impacts, tolerance, adaptation, and mitigation of heat stress on wheat under changing climates. Int. J. Mol. Sci. 23, 2838. doi: 10.3390/ijms23052838 35269980 PMC8911405

[B77] Yan J. K. Zhang S. Q. (2017). Effects of dwarfing genes on water use efficiency of bread wheat. Front. Agri. Sci. Eng. 4, 126–134. doi: 10.15302/J-FASE-2017134

[B78] Yin J. L. Fang Z. W. Sun C. Zhang P. Zhang X. Lu C.. (2018). Rapid identification of a stripe rust resistant gene in a space-induced wheat mutant using specific locus amplified fragment (SLAF) sequencing. Sci. Rep. 8, 3086. doi: 10.1038/s41598-018-21489-5 29449594 PMC5814476

[B79] Zhang Z. B. Shan L. (2000). Background analysis of genes controling water use efficiency of triticum. Acta Genet. Sinica. 27, 240–246. 10887696

[B80] Zhang Y. Wang L. Xin H. Li D. Ma C. Ding X.. (2013). Construction of a high-density genetic map for sesame based on large scale marker development by specific length amplified fragment (SLAF) sequencing. BMC Plant Biol. 13, 141. doi: 10.1186/1471-2229-13-141 24060091 PMC3852768

[B81] Zhang X. J. Yang Y. Li F. H. Xiang J. H. Liu D. Y. (2020). Construction of a high-density SNP genetic map for pacific white shrimp (Litopenaeus vannamei). Front. Genet 11, 571880. doi: 10.3389/fgene.2020.571880 33193676 PMC7541944

[B82] Zhu W. Y. Huang L. Chen L. Yang J. T. Wu J. N. Qu M. L.. (2016). A high-density genetic linkage map for cucumber (cucumis sativus l.): Based on specific length amplified fragment (SLAF) sequencing and QTL analysis of fruit traits in cucumber. Front. Plant Sci. 7. doi: 10.3389/fpls.2016.00437 PMC4835494 27148281

[B83] Zhu X. K. Sheng H. J. Gu J. Zhang R. Li C. Y. (2005). Primary study on application of SPAD value to estimate chlorophyll and nitrogen content in wheat leaves. J. Triticeae Crops. 25, 46–50. Available at: https://en.cnki.com.cn/Article_en/CJFDTOTALMLZW200502011.htm.

