# Supplementary material for: A pleiotropic QTL increased economic water use efficiency in bread wheat (Triticum aestivum L.)
Source: Front Plant Sci. 2023 Jan 4;13:1067590. doi: 10.3389/fpls.2022.1067590 (PMC9879270; doi:10.3389/fpls.2022.1067590)
Supplement: Supplementary file 1 [file Presentation_1.pdf]

A pleiotropic QTL increased economic water use efficiency in bread wheat (*Triticum aestivum* L.)

*Jian Hui, Haibo Bai, Xuelian Lyu, Sishuang Ma, Xiaojun Chen, Shuhua Li**

Correspondence: Shuhua Li, email: shuhua.l@163.com

SUPPLEMENTARY TABLES

Table S1 | Climatic characteristics of experimental sites in wheat growing season.

Site	Month	Maximum temperature (℃)	Minimum temperature (℃)	Rainfall	Characteristic
Yongning (106°14' E, 38°14' N, altitude of 1144m)	February	5.2	-8.1	4.6	i. Arid continental climate in the middle temperate zone of Ningxia plain. ii. Irrigated agriculture.
	March	15.5	1.2	2.3	
	April	22.3	7.3	4.0	
	May	27.1	13.6	2.0	
	June	31.2	18.4	14.9	
	July	33.4	20.3	17.2	
Yuanzhou (106°14' E, 36°09' N, altitude of 1567m)	February	3.2	-7.9	4.0	i. Semi arid continental climate in warm temperate zone of the loess plateau. ii. Rain-fed agriculture.
	March	11.2	-1.3	11.8	
	April	17.4	4.3	24.9	
	May	21.5	8.9	43.8	
	June	25.7	14.3	61.8	
	July	26.4	16.1	88.8	

Table S2 | Field irrigation amount of wheat in Ningxia.

Site	Irrigation treatment	Environment				Irrigation volume ($hm^3 \cdot ha^{-1}$)				Average soil humidity at 2 m depth (%)	
		2017	2018	2019	2020	tillering stage	Jointing stage	Heading stage	Filling stage	Before sowing	At harvest
Yongning	T1	EF1	EG1	EH1	EI1	1200	900	900	600	18.9	15.3
Yongning	T2	EF2	EG2	EH2	EI2	1200	900			18.9	13.2
Yuanzhou	T3	EF3	EG3	EH3	EI3					12.3	9.4

Table S3 | Traits measured for RIL population in 2017-2020 and their time of measurement each season.

	Traits	Abbreviate	Unit	Time
		Agronomic Traits		
1	Plant height	PH	cm	Before harvest
2	Fertile spikelet number per spike	FSN		After harvest
3	Number of grain per spike	GNS		After harvest
4	Spike number per unit area	SNUA	thousand·hm^{-2}	After harvest
5	Grain weight per spike	GWS	g	After harvest
6	Kilo-grain weight	KGW	g	After harvest
7	Grain yield per unit area	GYUA	t·hm^{-2}	After harvest
		Physiological Traits		
8	Leaf chlorophyll content	LCC		15 days after flowering
9	Canopy temperature	CT	℃	15 days after flowering
10	Carbon isotope discrimination	CID	%	After harvest
11	Water use efficiency of economic yield	WUE	kg·hm^{-2}·mm^{-1}	After harvest

.

Table S4 | QTL of water use efficiency of economic yield and its correlation traits in RIL mapping population.

QTL	Position (cM)	Chr. Distance (cM)	Relative Position	Left Marker	Right Marker	Left CI	Right CI	LOD	PVE (%)	Add.
QLcc.acn-1A(EG1)	20.9	95.0	22.0	*Marker11770808*	*Marker12356393*	20.2	22.4	7.2	19.7	-1.8
QGws.acn-1A(EF1)	31.0	95.0	32.6	*Marker11834380*	*Marker11519294*	29.8	31.4	3.8	9.0	-0.1
QPh.acn-1A.1(EF1)	12.1	95.0	12.7	*Marker11502188*	*Marker11932266*	11.4	12.6	2.7	6.2	-1.4
QPh.acn-1A.2(EG2)	85.4	95.0	89.9	*Marker11447863*	*Marker11489685*	85.3	86.6	4.0	6.9	-3.1
QSnua.acn-1A(EH3)	44.0	95.0	46.3	*Marker12086329*	*Marker11673876*	43.5	44.5	4.0	10.2	0.9
QWue.acn-1A(EF2)	24.3	95.0	25.6	*Marker11761559*	*Marker12240804*	24.3	24.7	4.6	12.1	-0.9
QLcc.acn-1B(EH1)	16.1	58.2	27.7	*Marker16203135*	*Marker16598300*	16.1	16.6	4.6	15.5	-1.5
QCt.acn-1B.1(EH1)	21.2	58.2	36.5	*Marker16100848*	*Marker16119088*	21.1	21.9	2.9	9.9	0.3
QCt.acn-1B.2(EH2)	21.9	58.2	37.7	*Marker16980457*	*Marker15983910*	21.9	23.7	9.8	23.0	0.5
QFsn.acn-1B(EF2)	32.4	58.2	55.7	*Marker16296023*	*Marker16732307*	32.0	32.9	3.7	4.8	0.5
QGns.acn-1B(EG3)	34.5	58.2	59.3	*Marker16284139*	*Marker16325705*	34.3	34.7	2.7	8.4	-1.5
QPh.acn-1B.1(EI1)	11.6	58.2	19.9	*Marker16597737*	*Marker16557679*	9.9	11.7	6.1	11.7	3.7
QPh.acn-1B.2(EF1)	21.9	58.2	37.7	*Marker16980457*	*Marker15983910*	21.9	25.3	4.9	11.7	1.9
QPh.acn-1B.3(EF2)	33.4	58.2	57.4	*Marker16162692*	*Marker16284139*	32.9	34.3	4.1	11.6	4.0
QPh.acn-1B.4(EG1)	35.2	58.2	60.5	*Marker17073346*	*Marker16113389*	35.2	35.6	3.6	8.5	3.0
QPh.acn-1B.5(EG2)	35.5	58.2	61.0	*Marker17073346*	*Marker16113389*	35.2	35.6	6.4	11.3	4.0
QSnua.acn-1B(EG1)	38.0	58.2	65.3	*Marker16707853*	*Marker15976961*	36.5	40.5	3.2	5.9	2.3
QWue.acn-1B(EI2)	5.6	58.2	9.6	*Marker16653992*	*Marker16266263*	5.3	5.7	4.4	5.7	0.6
QCid.acn-1D(EH3)	10.0	36.3	27.6	*Marker1654961*	*Marker1546145*	7.5	10.5	2.8	9.0	0.2

QFsn.acn-1D(EH1)	36.0	36.3	99.3	*Marker1612405*	*Marker1989643*	34.2	36.2	2.8	6.6	0.3
QGws.acn-1D(EF2)	10.2	36.3	28.1	*Marker1546145*	*Marker2001639*	7.4	10.6	2.9	12.2	0.1
QKgw.acn-1D(EI2)	14.2	36.3	39.2	*Marker2021337*	*Marker2029278*	13.9	15.4	2.6	8.8	1.3
QGns.acn-1D(EH1)	10.2	36.3	28.1	*Marker1546145*	*Marker2001639*	8.4	10.6	3.7	12.6	1.4
QSnua.acn-1D(EF2)	10.0	36.3	27.6	*Marker1654961*	*Marker1546145*	8.5	10.5	4.1	11.8	-1.6
QWue.acn-1D(EG3)	8.4	36.3	23.2	*Marker1654961*	*Marker1546145*	7.3	10.2	3.5	11.5	-0.6
QLcc.acn-2A(EH1)	0.8	75.4	1.1	*Marker21915904*	*Marker21663654*	0.8	2.5	3.3	10.9	1.3
QCid.acn-2A(EG2)	0.2	75.4	0.3	*Marker21071005*	*Marker21943117*	0.0	0.7	3.1	7.2	0.3
QCt.acn-2A(EG2)	31.8	75.4	42.2	*Marker20995945*	*Marker21775285*	31.5	32.1	4.3	14.8	0.7
QFsn.acn-2A.1(EG2)	39.8	75.4	52.8	*Marker21582916*	*Marker21842660*	38.7	39.9	3.8	9.8	-0.5
QFsn.acn-2A.2(EI1)	56.5	75.4	74.9	*Marker21053743*	*Marker20840880*	55.4	56.6	3.7	13.9	-0.5
QFsn.acn-2A.3(EI3)	60.3	75.4	80.0	*Marker21599358*	*Marker21170179*	60.2	62.2	4.1	13.6	-0.5
QFsn.acn-3A(EG3)	27.0	67.8	39.8	*Marker3810100*	*Marker3888343*	26.8	27.8	14.6	28.4	1.0
QGns.acn-2A(EG2)	34.3	75.4	45.5	*Marker20977264*	*Marker21924921*	33.3	35.0	4.9	16.0	-2.7
QPh.acn-2A(EF3)	69.1	75.4	91.7	*Marker20950622*	*Marker21494218*	67.5	70.9	5.1	17.2	3.3
QSnua.acn-2A(EH3)	66.0	75.4	87.5	*Marker21899367*	*Marker20697058*	65.5	66.5	3.0	7.5	-0.8
QWue.acn-2A(EH3)	57.8	75.4	76.7	*Marker20696676*	*Marker21165044*	57.4	58.6	2.7	9.2	-0.3
QPh.acn-2D.1(EH2)	9.6	30.4	31.6	*Marker7490790*	*Marker7800537*	9.1	10.0	10.0	4.6	4.5
QLcc.acn-2B(EI2)	73.1	126.0	92.1	Marker352143	Marker762683	92.0	92.4	5.5	21.5	1.4
QCid.acn-2B.1(EG2)	72.2	126.0	91.0	Marker650772	Marker580039	90.5	91.5	7.7	27.1	0.5
QCid.acn-2B.2(EF1)	72.7	126.0	91.6	Marker650772	Marker580039	90.4	91.6	9.9	24.8	0.4
QCid.acn-2B.3(EG3)	73.1	126.0	92.1	Marker352143	Marker762683	92.0	92.3	8.2	27.2	0.3
QCid.acn-2B.4(EG1)	74.0	126.0	93.3	Marker1025623	Marker1330209	93.0	94.0	8.8	18.0	0.3
QCt.acn-2B.1(EG1)	68.1	126.0	85.8	Marker1138705	Marker1289035	83.7	88.8	8.1	17.8	-0.5
QCt.acn-2B.2(EG1)	88.7	126.0	111.7	Marker1185416	Marker354231	110.7	111.7	3.7	6.2	-0.3

QFsn.acn-2B(EI2)	11.7	126.0	14.7	Marker968768	Marker974608	14.4	15.1	4.3	12.7	-0.4
QGws.acn-2B(EG3)	73.1	126.0	92.1	Marker352143	Marker762683	92.0	92.4	5.4	17.0	0.1
QGws.acn-2B(EI2)	73.1	126.0	92.1	Marker352143	Marker762683	92.0	92.4	9.2	28.5	0.2
QKgw.acn-2B(EG2)	73.1	126.0	92.1	Marker352143	Marker762683	92.0	92.3	14.8	19.6	4.0
QKgw.acn-2B(EI2)	73.1	126.0	92.1	Marker352143	Marker762683	92.0	92.4	4.7	16.8	1.9
QGns.acn-2B(EF2)	72.2	126.0	91.0	Marker650772	Marker580039	90.5	91.5	6.3	20.9	2.5
QGns.acn-2B(EI1)	72.2	126.0	91.0	Marker650772	Marker580039	90.5	91.5	6.0	15.7	2.8
QPh.acn-2B.1(EH1)	72.5	126.0	91.3	Marker650772	Marker580039	90.4	91.6	5.9	13.5	-4.3
QPh.acn-2B.2(EG3)	72.7	126.0	91.6	Marker650772	Marker580039	90.5	91.6	6.9	20.9	-4.6
QPh.acn-2B.2(EH3)	72.7	126.0	91.6	Marker650772	Marker580039	90.6	91.6	5.8	16.0	-4.1
QPh.acn-2B.2(EI3)	72.7	126.0	91.6	Marker650772	Marker580039	90.7	91.6	12.0	12.2	-4.8
QSnua.acn-2B(EG2)	41.3	126.0	52.0	Marker800553	Marker324748	49.5	53.5	3.1	10.9	2.5
QWue.acn-2B.1(EI1)	71.8	126.0	15.9	Marker1292490	Marker587273	15.6	16.0	3.0	10.8	0.4
QWue.acn-2B.2(EG1)	71.8	126.0	90.5	Marker650772	Marker580039	90.4	91.6	4.7	15.6	0.9
QWue.acn-2B.2(EH1)	72.2	126.0	90.5	Marker650772	Marker580039	90.4	91.6	7.0	22.8	0.8
QWue.acn-2B.3(EI2)	73.1	126.0	91.0	Marker650772	Marker580039	90.5	91.5	4.0	26.3	1.5
QWue.acn-2B.4(EI3)	75.6	126.0	92.1	Marker352143	Marker762683	92.0	92.4	8.4	26.1	-0.8
QWue.acn-2B.5(EG3)	73.1	126.0	95.3	Marker1025623	Marker1330209	94.1	96.2	5.0	16.0	-0.7
QPh.acn-2D.2(EH2)	10.8	30.4	35.5	*Marker7912561*	*Marker7377329*	10.6	10.9	19.9	11.2	-7.1
QLcc.acn-3B.1(EH2)	48.4	92.2	52.5	*Marker18320736*	*Marker17646285*	47.4	50.2	4.3	8.9	-1.6
QLcc.acn-3B.2(EG1)	77.7	92.2	84.3	*Marker18233279*	*Marker17947919*	76.8	78.1	3.8	9.2	1.2
QCid.acn-3B(EG1)	87.0	92.2	94.4	*Marker17673791*	*Marker18104409*	86.2	87.8	5.0	9.9	-0.2
QFsn.acn-3B.1(EG2)	4.8	92.2	5.2	*Marker17473745*	*Marker18134551*	4.5	4.9	4.7	12.5	0.5
QFsn.acn-3B.2(EH2)	10.5	92.2	11.4	*Marker17607502*	*Marker17123684*	10.3	10.7	9.8	22.5	0.9
QGws.acn-3B(EF1)	10.3	92.2	11.2	*Marker17399841*	*Marker17607502*	10.1	10.7	7.3	23.3	0.2

QGws.acn-3B(EH1)	10.3	92.2	11.2	*Marker17399841*	*Marker17607502*	10.1	10.8	6.6	20.2	0.2
QKgw.acn-3B(EF1)	66.8	92.2	72.4	*Marker18418003*	*Marker17699667*	65.6	68.1	3.3	8.6	1.5
QGns.acn-3B(EH2)	10.4	92.2	11.3	*Marker17399841*	*Marker17607502*	10.1	10.7	5.1	16.9	2.6
QSnua.acn-3B.1(EI2)	45.0	92.2	48.8	*Marker17276163*	*Marker18016667*	44.5	45.5	5.8	17.0	2.1
QSnua.acn-3B.2(EH1)	86.0	92.2	93.3	*Marker17476916*	*Marker17749993*	85.5	86.5	3.0	11.9	-1.9
QLcc.acn-3D(EF2)	0.2	70.1	0.3	*Marker2942826*	*Marker3134969*	0.0	0.9	2.8	8.9	-1.3
QCid.acn-3D(EF2)	13.4	70.1	19.1	*Marker2841475*	*Marker3098843*	13.4	14.1	7.5	16.2	-0.2
QFsn.acn-3D(EI2)	5.7	70.1	8.1	*Marker2403061*	*Marker2465407*	5.7	6.5	2.6	6.9	0.3
QGws.acn-3D(EG2)	25.5	70.1	36.4	*Marker2887002*	*Marker3141685*	25.1	26.4	3.6	6.7	0.1
QKgw.acn-3D.1(EG2)	18.8	70.1	26.8	*Marker2477522*	*Marker3105738*	16.4	19.0	7.9	9.2	2.7
QKgw.acn-3D.2(EH1)	57.5	70.1	82.0	*Marker2582315*	*Marker2825857*	55.0	58.3	2.6	8.8	-1.9
QPh.acn-3D(EF2)	3.7	70.1	5.3	*Marker2999220*	*Marker2789259*	3.6	4.5	2.7	7.4	-3.3
QSnua.acn-3D(EG1)	6.0	70.1	8.6	*Marker2403061*	*Marker2465407*	5.5	6.5	6.8	13.4	-3.5
QLcc.acn-4A(EG2)	57.1	103.0	55.4	*Marker6199317*	*Marker6016220*	55.8	60.0	6.1	14.0	-1.6
QGws.acn-4A(EF3)	61.6	103.0	59.8	*Marker6199317*	*Marker6016220*	58.1	61.6	2.5	9.5	0.1
QCid.acn-4B.1(EF2)	39.2	78.4	50.0	*Marker9231835*	*Marker9772027*	38.2	39.3	3.6	7.2	-0.2
QCid.acn-4B.2(EH3)	64.0	78.4	81.6	*Marker9038958*	*Marker10124144*	63.5	64.5	3.3	10.4	0.2
QCt.acn-4B(EH2)	57.0	78.4	72.7	*Marker9131610*	*Marker10063287*	56.9	57.9	4.6	9.8	0.3
QFsn.acn-4B(EF3)	78.4	78.4	100.0	*Marker9305419*	*Marker10021999*	77.6	78.4	2.6	3.3	0.3
QKgw.acn-4B(EH2)	64.3	78.4	82.0	*Marker9038958*	*Marker10124144*	63.9	64.3	4.5	15.7	-2.3
QPh.acn-4B.1(EG2)	26.9	78.4	34.3	*Marker10034433*	*Marker10212592*	26.7	27.6	7.7	14.0	-4.5
QPh.acn-4B.2(EG1)	27.1	78.4	34.6	*Marker10212592*	*Marker9542811*	26.7	27.9	7.7	20.1	-4.7
QPh.acn-4B.2(EH2)	27.1	78.4	34.6	*Marker10212592*	*Marker9542811*	26.7	28.0	4.5	1.9	-2.9
QWue.acn-4B(EI1)	55.4	78.4	70.7	*Marker10244362*	*Marker9271444*	55.2	55.6	4.8	17.6	-0.5
QLcc.acn-4D(EF1)	77.4	77.5	99.9	*Marker23370125*	*Marker23773620*	76.7	77.4	3.5	11.3	2.3

QCid.acn-4D.1(EG2)	47.9	77.5	61.8	*Marker23494510*	*Marker23326071*	46.5	48.8	4.3	10.3	-0.3
QCid.acn-4D.2(EH1)	52.0	77.5	67.1	*Marker23326071*	*Marker23357468*	49.5	54.5	4.0	6.3	0.2
QCt.acn-4D.1(EF2)	0.4	77.5	0.5	*Marker23409443*	*Marker23964440*	0.3	0.5	3.6	10.9	0.3
QCt.acn-4D.2(EF1)	1.7	77.5	2.2	*Marker23519973*	*Marker23821505*	1.7	2.4	5.0	14.2	0.4
QCt.acn-4D.3(EG1)	49.4	77.5	63.8	*Marker23326071*	*Marker23357468*	48.0	51.3	9.7	19.1	0.6
QGws.acn-4D(EG1)	13.0	77.5	16.8	*Marker23408404*	*Marker23909431*	12.2	13.5	2.8	9.4	0.1
QPh.acn-4D.1(EF1)	0.4	77.5	0.5	*Marker23409443*	*Marker23964440*	0.2	0.5	4.9	11.9	1.9
QPh.acn-4D.1(EG3)	0.4	77.5	0.5	*Marker23409443*	*Marker23964440*	0.2	0.5	3.1	8.8	2.9
QPh.acn-4D.1(EH1)	0.4	77.5	0.5	*Marker23409443*	*Marker23964440*	0.3	0.5	7.0	16.0	4.5
QPh.acn-4D.1(EH3)	0.4	77.5	0.5	*Marker23409443*	*Marker23964440*	0.3	0.5	5.0	13.8	3.7
QPh.acn-4D.1(EI3)	0.4	77.5	0.5	*Marker23409443*	*Marker23964440*	0.3	0.5	11.3	11.4	4.5
QPh.acn-4D.2(EH2)	40.2	77.5	51.9	*Marker23792768*	*Marker23762812*	40.1	40.5	4.3	1.9	-2.9
QPh.acn-4D.3(EI3)	40.9	77.5	52.8	*Marker23730549*	*Marker23600322*	40.6	40.9	18.3	20.9	-6.2
QWue.acn-4D(EF1)	1.7	77.5	2.2	*Marker23519973*	*Marker23821505*	1.7	2.5	3.8	11.6	-0.7
QGws.acn-5A(EI3)	102.4	114.2	89.6	*Marker23037121*	*Marker22613531*	99.1	103.1	3.3	11.6	-0.1
QLcc.acn-5B(EG2)	89.3	102.5	87.1	*Marker19615603*	*Marker19393245*	88.9	89.6	3.1	6.8	-1.1
QCid.acn-5B.1(EF2)	4.3	102.5	4.2	*Marker19134665*	*Marker19168821*	4.1	4.9	7.3	15.5	0.2
QCid.acn-5B.2(EH1)	5.0	102.5	4.9	*Marker18700897*	*Marker19036876*	4.5	5.5	5.2	7.2	0.2
QCid.acn-5B.3(EG1)	19.2	102.5	18.7	*Marker19165435*	*Marker19028090*	17.6	19.3	3.1	5.8	0.2
QFsn.acn-5B(EG3)	89.6	102.5	87.4	*Marker19615603*	*Marker19393245*	89.2	90.0	3.7	5.3	-0.4
QGws.acn-5B.1(EG3)	82.7	102.5	80.7	*Marker19571747*	*Marker18796346*	82.6	82.9	3.5	11.2	-0.1
QGws.acn-5B.2(EI3)	86.8	102.5	84.7	*Marker19356279*	*Marker19059252*	86.3	86.9	2.7	8.6	-0.1
QGns.acn-5B.1(EG3)	82.7	102.5	80.7	*Marker19571747*	*Marker18796346*	82.6	82.9	4.3	13.2	-1.9
QGns.acn-5B.2(EI3)	86.6	102.5	84.5	*Marker19209690*	*Marker19356279*	86.3	86.9	2.7	9.2	-1.6
QSnua.acn-5B(EH3)	12.0	102.5	11.7	*Marker18480586*	*Marker19509490*	6.5	13.5	4.4	13.6	1.0

QCid.acn-5D.1(EF1)	14.3	96.5	14.8	*Marker14748663*	*Marker14044565*	13.5	14.8	2.6	5.8	0.2
QCid.acn-5D.2(EF2)	50.7	96.5	52.6	*Marker14442564*	*Marker14574143*	50.2	51.0	3.3	6.6	-0.2
QCt.acn-5D.1(EH2)	51.0	96.5	52.9	*Marker14442564*	*Marker14574143*	50.7	51.0	2.9	6.3	-0.3
QCt.acn-5D.2(EH2)	68.9	96.5	71.4	*Marker13954176*	*Marker14780393*	67.4	69.9	2.9	6.6	-0.3
QPh.acn-5D(EH1)	69.3	96.5	71.8	*Marker13954176*	*Marker14780393*	68.3	70.0	4.5	9.7	-3.5
QPh.acn-5D(EI1)	69.3	96.5	71.8	*Marker13954176*	*Marker14780393*	68.4	70.0	4.5	8.4	-3.1
QKgw.acn-6A(EG2)	15.7	68.2	23.0	*Marker19704933*	*Marker20376281*	15.1	16.4	2.9	3.1	1.5
QLcc.acn-6B(EH2)	28.4	38.9	72.9	*Marker13468817*	*Marker13187343*	27.7	28.8	3.2	6.2	1.3
QPh.acn-6B(EH3)	28.4	38.9	72.9	*Marker13468817*	*Marker13187343*	27.7	28.8	2.7	6.9	-2.6
QLcc.acn-6D(EH2)	3.2	55.7	5.7	*Marker8720360*	*Marker8293984*	2.6	4.0	4.4	9.1	-1.6
QCt.acn-6D(EF1)	16.1	55.7	28.9	*Marker8668903*	*Marker8865441*	15.7	16.2	3.7	10.4	-0.3
QFsn.acn-6D(EF1)	35.1	55.7	63.0	*Marker8939620*	*Marker8600735*	34.0	36.2	4.0	13.5	-0.5
QGws.acn-6D.1(EG2)	11.4	55.7	20.5	*Marker8881601*	*Marker8894622*	10.5	12.7	3.5	8.2	-0.1
QGws.acn-6D.2(EF2)	20.2	55.7	36.2	*Marker8348096*	*Marker8740286*	20.2	20.6	2.9	12.7	-0.1
QKgw.acn-6D(EH1)	3.3	55.7	5.9	*Marker8293984*	*Marker8661156*	2.7	4.2	4.1	14.2	-2.5
QGns.acn-6D(EH1)	13.4	55.7	24.0	*Marker8869039*	*Marker8948349*	13.4	14.2	2.6	8.5	-1.1
QSnua.acn-6D.1(EF2)	3.0	55.7	5.4	*Marker8720360*	*Marker8293984*	2.5	4.5	6.2	19.7	2.1
QSnua.acn-6D.2(EF1)	20.0	55.7	35.9	*Marker8591667*	*Marker8954952*	19.5	20.5	3.0	10.7	1.8
QSnua.acn-6D.4(EH2)	36.0	55.7	64.6	*Marker8939620*	*Marker8600735*	35.5	36.5	2.7	8.8	2.0
QSnua.acn-6D.3(EI1)	35.0	55.7	62.8	*Marker8451613*	*Marker8939620*	33.5	36.5	2.6	8.8	1.1
QWue.acn-6D(EF2)	16.1	55.7	28.9	*Marker8668903*	*Marker8865441*	15.5	16.2	2.6	6.8	0.7
QLcc.acn-7A.1(EH2)	5.8	88.2	6.6	*Marker10785678*	*Marker11084787*	4.9	6.0	4.6	9.1	-1.6
QLcc.acn-7A.2(EF2)	69.4	88.2	78.7	*Marker10626252*	*Marker11227369*	68.6	69.4	3.5	10.6	-1.4
QCt.acn-7A.1(EF3)	6.7	88.2	7.6	*Marker10671886*	*Marker10405781*	6.0	6.8	2.8	9.8	-0.5
QCt.acn-7A.2(EI2)	13.6	88.2	15.4	*Marker10553156*	*Marker10352245*	13.3	13.7	2.6	9.0	0.2

QCt.acn-7A.3(EI2)	62.2	88.2	70.5	*Marker10589014*	*Marker11379340*	62.0	62.2	4.8	17.0	-0.2
QFsn.acn-7A.1(EI1)	60.5	88.2	68.6	*Marker11226905*	*Marker10797715*	59.9	61.8	5.4	20.8	0.7
QFsn.acn-7A.2(EG1)	63.0	88.2	71.4	*Marker11379340*	*Marker10631500*	62.2	63.0	5.6	18.2	0.9
QFsn.acn-7A.3(EH3)	63.1	88.2	71.5	*Marker11427358*	*Marker11140845*	63.0	63.4	3.8	13.0	0.5
QFsn.acn-7A.4(EI2)	64.9	88.2	73.6	*Marker10594544*	*Marker11143402*	64.3	64.9	5.3	15.3	0.5
QFsn.acn-7A.5(EG2)	87.2	88.2	98.9	*Marker10486767*	*Marker11247509*	87.0	87.8	2.5	6.7	0.4
QGws.acn-7A(EF2)	5.6	88.2	6.3	*Marker10785678*	*Marker11084787*	4.9	5.8	2.7	11.3	-0.1
QKgw.acn-7A(EF1)	6.7	88.2	7.6	*Marker10671886*	*Marker10405781*	6.3	6.8	7.0	19.2	-2.3
QGns.acn-7A(EI2)	20.6	88.2	23.4	*Marker10444014*	*Marker10855375*	20.3	21.5	6.4	18.6	2.5
QPh.acn-7A(EH1)	8.4	88.2	9.5	*Marker10248726*	*Marker11293256*	8.4	8.8	2.7	5.6	2.7
QWue.acn-7A(EH3)	85.1	88.2	96.5	*Marker10460129*	*Marker11133470*	85.0	85.4	3.6	12.3	0.4
QCid.acn-7B(EH1)	11.0	89.5	12.3	*Marker4902069*	*Marker5533195*	10.5	12.5	4.0	5.4	0.2
QCt.acn-7B(EF2)	29.3	89.5	32.7	*Marker5621425*	*Marker5641116*	27.7	29.4	2.8	8.3	-0.3
QKgw.acn-7B(EG3)	57.5	89.5	64.2	*Marker5693377*	*Marker5405188*	57.4	57.7	5.7	17.4	2.4
QWue.acn-7B(EG2)	17.7	89.5	19.8	*Marker4757001*	*Marker4884071*	17.5	17.8	3.1	12.1	-0.7
QLcc.acn-7D(EF1)	107.6	121.4	88.7	*Marker15602355*	*Marker15432219*	104.7	111.5	2.5	8.6	-2.0
QCt.acn-7D(EI1)	95.7	121.4	78.8	*Marker15931560*	*Marker15116422*	95.4	96.5	2.7	9.4	0.2
QKgw.acn-7D(EG3)	96.4	121.4	79.4	*Marker15931560*	*Marker15116422*	95.6	96.5	2.8	8.0	-1.6
QWue.acn-7D(EG2)	99.4	121.4	81.9	*Marker15412018*	*Marker15256400*	97.8	101.7	3.3	13.2	-0.8
